# Ultrafast spectral hole burning reveals the distinct chromophores in eumelanin and their common photoresponse†

**DOI:** 10.1039/c9sc04527a

**Published:** 2019-12-18

**Authors:** Forrest R. Kohl, Christopher Grieco, Bern Kohler

**Affiliations:** Department of Chemistry and Biochemistry, The Ohio State University 100 West 18^th^ Avenue Columbus Ohio 43210 USA kohler.40@osu.edu +1-614-688-2635

## Abstract

Eumelanin, the brown-black pigment found in organisms from bacteria to humans, dissipates solar energy and prevents photochemical damage. While the structure of eumelanin is unclear, it is thought to consist of an extremely heterogeneous collection of chromophores that absorb from the UV to the infrared, additively producing its remarkably broad absorption spectrum. However, the chromophores responsible for absorption by eumelanin and their excited state decay pathways remain highly uncertain. Using femtosecond broadband transient absorption spectroscopy, we address the excited state behavior of chromophore subsets that make up a synthetic eumelanin, DOPA melanin, and probe the heterogeneity of its chromophores. Tuning the excitation light over more than an octave from the UV to the visible and probing with the broadest spectral window used to study any form of melanin to date enable the detection of spectral holes with a linewidth of 0.6 eV that track the excitation wavelength. Transient spectral hole burning is a manifestation of extreme chemical heterogeneity, yet exciting these diverse chromophores unexpectedly produces a common photoinduced absorption spectrum and similar kinetics. This common photoresponse is assigned to the ultrafast formation of immobile charge transfer excitons that decay locally and that are formed among graphene-like chromophores in less than 200 fs. Raman spectroscopy reveals that chromophore heterogeneity in DOPA melanin arises from different sized domains of sp^2^-hybridized carbon and nitrogen atoms. Furthermore, we identify for the first time striking parallels between the excited state dynamics of eumelanin and disordered carbon nanomaterials, suggesting that they share common structural attributes.

## Abstract 2

Seeing the colors in black: ultrafast transient hole burning spectroscopy reveals the absorption properties of discrete chromophores and their interactions in the skin pigment eumelanin.

## Graphical abstract

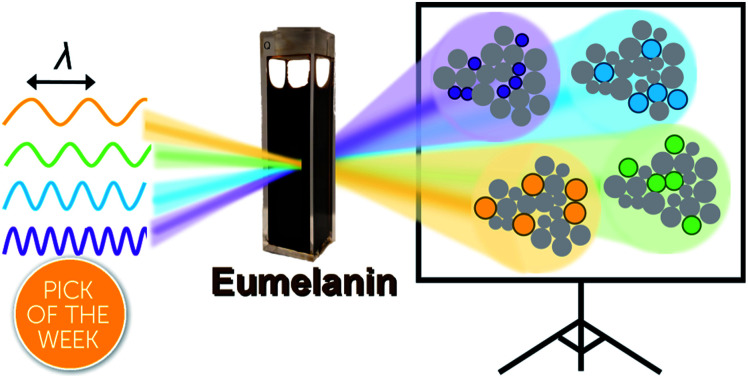

## Introduction

Melanin is most familiar as a family of pigments that color human skin and hair, but melanins are found throughout the tree of life in organisms ranging from bacteria and fungi to plants and mammals. The brown/black form known as eumelanin absorbs UV, visible, and near infrared (NIR) light and efficiently dissipates the light energy as heat.^1^ In the skin, eumelanin acts as a natural sunscreen that protects DNA from photodamage, but also serves other functions such as sequestering metal ions and scavenging damaging radicals.^1^ Indeed, their wide taxonomic distribution implies that melanins are versatile, multifunctional materials of great evolutionary importance. Because the structures of all melanins, including eumelanin, remain obscure, fundamental understanding of photoprotection and other properties is lacking. Even the most basic question that might be asked about a pigment—*what gives it its color?*—still cannot be answered definitively for any melanin despite decades of study.

**Fig. 1:**
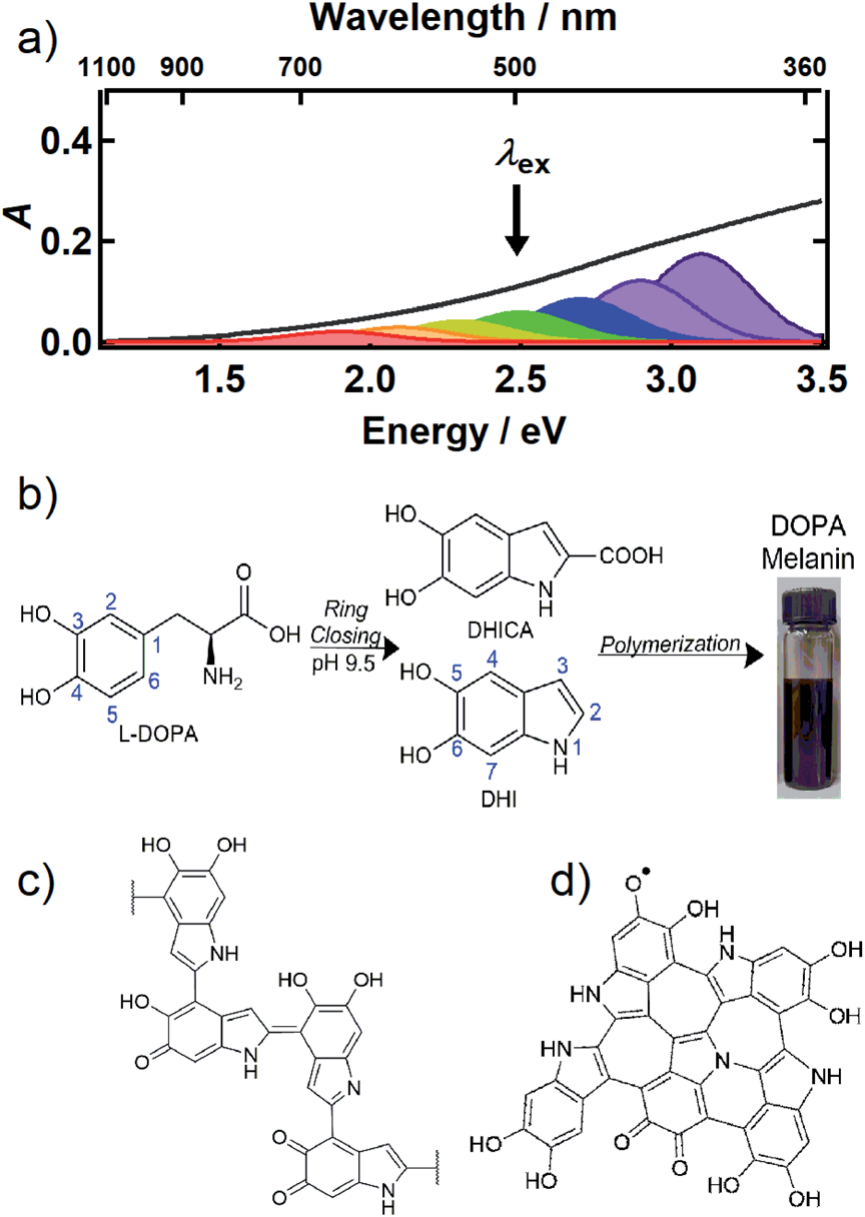
(a) UV-Vis-NIR absorption spectrum of an aqueous solution of DOPA melanin (black trace). The colored Gaussian functions illustrate hypothetical, distinct chromophores, some of which can be selectively excited by a given excitation wavelength (black arrow). (b) Synthesis of DOPA melanin from l-DOPA, showing the DHI and DHICA intermediates. Typical structural motifs proposed for eumelanin are shown in (c) and (d): (c) irregular, possibly branched macromolecule, and (d) two-dimensional oligomeric protomolecule (adapted from ref. 59).

The UV-Vis-NIR absorption spectrum of an aqueous solution of the synthetic eumelanin, known as DOPA melanin, is broad and featureless (Fig. 1a) and increases monotonically toward higher photon energy. Virtually the same spectrum is measured for the naturally occurring eumelanin isolated from the cuttlefish, *Sepia officinalis* (see Fig. 1 in ref. 2). Synthetic eumelanin polymers are convenient surrogates for natural melanin as they are thought to contain the same covalently and noncovalently interacting chemical units found in the natural material, but they do not suffer from protein contamination. They are readily synthesized by oxidative polymerization under very mild conditions starting with aqueous solutions of compounds such as tyrosine, l-DOPA, or dopamine (Fig. 1b). These precursors react to form the intermediates 5,6-dihydroxyindole (DHI) and 5,6-dihydroxyindole-2-carboxylic acid (DHICA) (see Fig. 1b), which polymerize to form the final eumelanin product. However, the final structure of eumelanin and even the chemical composition of the subunits responsible for light absorption are obscure.

Despite uncertainty in its structure, there is considerable agreement today that eumelanin is made up of a collection of heterogeneous subunits characterized by narrower absorption spectra. This ‘chemical disorder model’^3,4^ explains the heterogeneity as arising from a diversity in chemical structures, which differ in conjugation length, number of aromatic rings, and the redox state of the absorbing subunits (hydroquinone, semiquinone, quinone methide, and quinone forms).^5–7^ The center wavelengths of their lowest-energy transitions are distributed throughout the broad absorption spectrum of eumelanin, as illustrated by the colored Gaussians in Fig. 1a. For clarity of illustration, only a small number of different kinds of absorbers are shown in the figure. Geometrical or molecular packing disorder may further contribute to dispersion in the possible transition energies through excitonic interactions.^8^

Eumelanin has been discussed most often as consisting either of irregular polymer chains (Fig. 1c), or layered nano-aggregates made of planar ‘protomolecules’ (Fig. 1d); the latter consist of conjugated five- and six-membered rings formed by oligomerization of small numbers of DHI and/or DHICA units in chemically distinct combinations that are layered in graphite-like stacks.^1^ While the chemical disorder model explains the absorption spectrum and excitation wavelength-dependent emission behavior of natural and synthetic eumelanins,^9,10^ it does not address the identity of the actual absorbers, how they are organized in eumelanin, or the extent to which they interact. Our aim in this study is to gain new insight into the diverse absorbing subunits of eumelanin, which we will refer to hereafter as *chromophores*. We will use this term regardless of whether the absorbers are discrete molecular units that form larger assemblies through noncovalent interactions or are merely domains within larger structures such as layered oligomers or polymer chains. We will also use chromophore to refer inclusively to absorbers, regardless of whether their maximum absorbance is in the visible, UV, or NIR.

The elusive molecular architecture of eumelanin has stymied efforts to understand its photoprotective mechanisms. The desire to uncover structure–function relationships in eumelanin has inspired studies of the DHI and DHICA precursors along with oligomers formed from them.^11–18^ This bottom-up approach has provided insight into the decay pathways of these putative eumelanin subunits, but the lack of agreement between their absorption spectra and that of eumelanin indicates that these studies cannot capture the full set of eumelanin chromophores. Additionally, these studies do not capture excitonic interactions between different kinds of chromophores, which are hypothesized to play a central role in the excited state dynamics of eumelanin.^8,19,20^ Thus, top-down studies are crucial for studying how heterogeneity affects the excited state dynamics of eumelanin. To this end, transient absorption^2,19–25^ and photoluminescence^10,17,26,27^ studies of natural and synthetic eumelanins have been carried out, but it has proven difficult to link dynamical observations with structural models.

Here, we investigate DOPA melanin by broadband femtosecond transient absorption (TA) spectroscopy as a function of excitation wavelength to gain insight into the deactivation pathways of the excited states formed by different subsets of chromophores. Taking this approach, we gain new insights into the nature and consequences of the electronic coupling among the chromophores of melanin, and into the role that heterogeneity plays in shaping the diverse functions of eumelanin, including solar energy dissipation. Even without knowledge of the chromophores that are excited, the timescales of deactivation provide important information about the nature of the excitations and their relaxation pathways. Additionally, because deactivation *via* excitation energy transfer (EET) or charge transfer (CT) depends sensitively on distance-dependent electronic couplings between chromophores, excited state dynamics necessarily encode information about structure when these decay pathways are present.^28^

Our work demonstrates that excitation wavelength-dependent TA spectroscopy is a practical method for top-down studies of melanins despite the challenges imposed by their heterogeneous makeup. By pumping and probing over a wider spectral region than in any previous melanin TA study, we show that photoexcitation induces a transient spectral hole centered about the excitation wavelength as it is tuned through the visible spectrum. These new results reconcile and extend past observations of ground state bleaching from TA spectroscopy made with narrower probe windows. They show that the absorption spectrum of eumelanin is broadened due to static chemical heterogeneity and they provide new insights into the dynamics and interactions among the chromophores. Additionally, our study reveals striking similarities in the excited state dynamics of eumelanin and disordered carbonaceous nanomaterials, suggesting that these diverse materials consist of common chromophores organized according to common structural motifs.

## Results and discussion

### Chromophore heterogeneity revealed by transient spectral hole burning

**Fig. 2:**
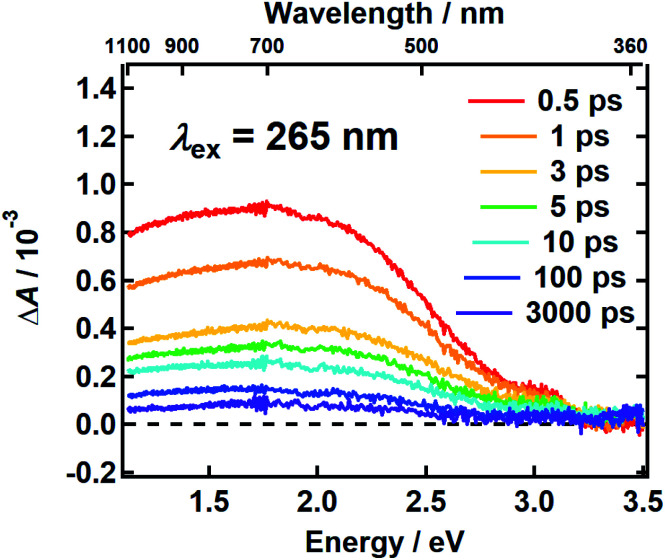
The transient absorption spectrum of DOPA melanin in aqueous solution excited at 265 nm at the indicated delay times *vs.* probe photon energy (bottom axis) and *vs.* probe wavelength (top axis). The spectrum was recorded using magic angle pump–probe polarizations.

It follows from the chemical disorder model that a pump pulse tuned to a specific wavelength (*e.g.*, black arrow in Fig. 1a) in the broad absorption spectrum of eumelanin will selectively excite a subset of chromophores. In our femtosecond TA measurements, the absorbance change (Δ*A*) in a broadband probe pulse measures the dynamics of this subset of chromophores as a function of the delay time between the pump and probe pulses. The broadband TA spectra recorded from a DOPA melanin solution excited at 265 nm are shown in Fig. 2. A nearly featureless broad band with a maximum near 1.8 eV is seen at each delay time. At probe energies greater than 2 eV, the spectrum falls rapidly, approaching zero near 3.3 eV. At all delay times, the spectral shape remains the same within experimental uncertainty (Fig. S1†) aside from very weak, negative signals seen at the highest probe energies at early times (also see Fig. S2a†). On the low energy side, the transient spectrum falls off more slowly and still has 85% of the maximum band amplitude at our lowest probe energy (1.1 eV).

The first TA spectrum for any melanin was reported by Ye and Simon,^21^ who excited *Sepia* melanin with 120 fs pulses at 303 nm. Their transient spectrum measured at 1 ps delay time shows positive signals everywhere between 500 and 800 nm in agreement with ours, but the spectrum in ref. 21, which was assembled from measurements made at a small number of discrete probe wavelengths, is more modulated in appearance with several peaks, which are not seen in Fig. 2. A more recent TA spectrum reported by Brunetti *et al.*^25^ for *Sepia* melanin in DMSO–methanol (20:1) excited at 350 nm appears to vary smoothly across the visible spectrum in agreement with ours. However, detailed comparison is not possible because the TA signals in ref. 25 are presented as a two-dimensional time–wavelength heat or color map. The maximum in our spectrum at ∼700 nm is in rough agreement with the maximum of 625 nm reported in ref. 25.

Assignments for the TA spectra in Fig. 2 will be discussed later, but we note here that the observed band does not resemble the TA spectrum of the DHI monomer, which has bands at 550 nm (2.25 eV) and 450 nm (2.76 eV).^12^ In fact, the amplitude of the transient spectrum of the DHI monomer in ref. 12 decreases with decreasing photon energy through the visible region, whereas the opposite behavior is seen for DOPA melanin. These differences suggest that the excited states in DHI and the eumelanin polymer differ in electronic character, and so we expect them to follow different excited state decay pathways.

**Fig. 3:**
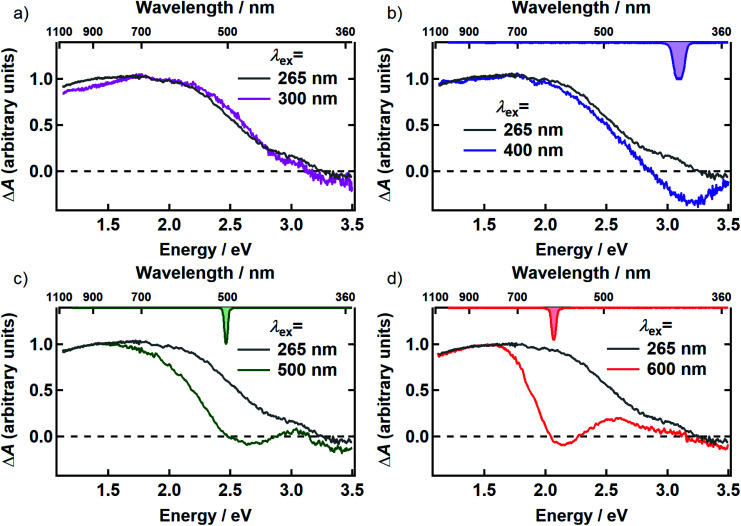
Comparison of the TA spectrum for 265 nm excitation (gray) with the TA spectra for (a) 300 nm (magenta) (b) 400 nm (purple) (c) 500 nm (green) and (d) 600 nm (red) excitation at 500 fs after excitation. The inverted spectrum of the excitation pulse is shown on the top axis in panels b–d. All transient spectra shown were acquired with perpendicular pump and probe polarizations.

In Fig. 3, the transient spectrum measured 500 fs after excitation at 265 nm (gray curve in panels a–d) is compared with the transient spectrum recorded at four different pump wavelengths at the same delay time (colored curves in Fig. 3). The two spectra in each panel were scaled to agree at wavelengths longer than 800 nm. Transient spectra recorded at later delay times (see Fig. S1 and S2†) are similar in shape to the spectrum recorded at 500 fs, showing that there is minimal spectral evolution regardless of pump wavelength. To minimize interference from pump scatter, the TA spectra in Fig. 3 and S2† were recorded with perpendicular pump and probe polarizations but similar spectra were observed with polarizations set to the magic angle (Fig. S3†).

After scaling, the spectrum excited at 300 nm agrees within experimental uncertainty with the one recorded at 265 nm (Fig. 3a). Both signals are positive (Δ*A* > 0) everywhere in the probe window, except in the near-UV where weak, negative signals are seen. In TA experiments, positive signal contributions at a given probe energy arise from photoinduced absorption (PA) by transient species created by the pump pulse such as excited states and photoproducts. Depletion due to photoexcitation, referred to as ground state bleaching (GSB), makes a negative signal contribution. Negative signal contributions can also arise from stimulated emission (SE) by excited states.

Although positive and negative TA signals are often referred to as PA or GSB signals, respectively, signals of either sign may contain contributions from PA, GSB, and SE when the bands responsible for these signals overlap. Thus, although a positive signal indicates that PA is definitely present, it does not rule out contributions from GSB or SE. Because DOPA melanin absorbs throughout our probe window, overlap between the PA and the GSB signals is to be expected. Hereafter, we will use the terms PA or GSB signals (SE is considered to be negligible as discussed below) to refer to the *contributions* made by each to the total Δ*A* signal. Our results allow these contributions to the broadband TA signals of eumelanin to be disentangled for the first time.

Compared to the TA spectrum recorded with a pump wavelength of 265 or 300 nm, excitation at the visible wavelengths shown in Fig. 3b–d ‘burns’ a broad dip, or spectral hole, centered about the pump wavelength. In panels b–d, the spectrum of the femtosecond excitation pulse is shown inverted on the upper horizontal axis. The spectral width of the hole is more than an order of magnitude greater than that of the femtosecond pump pulse at every pump wavelength where hole burning is observed. Away from the pump wavelength, the transient spectrum agrees well with the one recorded with 265 nm excitation. However, the departure of the visible signals from the one recorded at 265 nm at probe wavelengths on either side of the pump wavelength indicates that there are negative contributions (GSB or SE) to the TA signals even at wavelengths where the overall Δ*A* signals are positive.

**Fig. 4:**
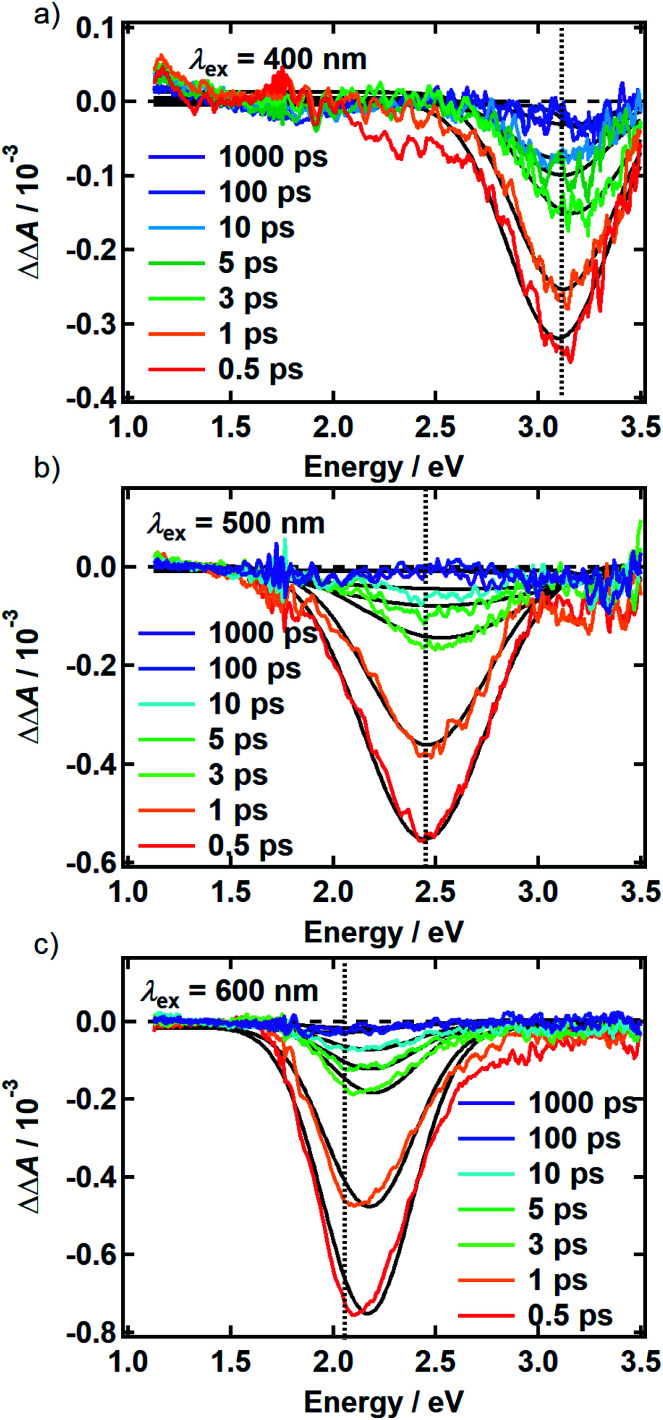
Time-resolved ground state bleach spectrum isolated from transient absorption spectra recorded using the perpendicular pump-probe polarizations as described in the text. The spectra are shown for (a) 400 nm, (b) 500 nm, and (c) 600 nm excitation. The photon energy of each pump pulse is indicated by the vertical dashed line. Solid black lines are the Gaussian best fits at each time point.

There is no evidence of SE in our TA experiments on DOPA melanin. SE can only be observed when the probe wavelength is longer than the pump wavelength, so hole burning seen at wavelengths to the blue of the pump wavelength cannot be due to SE and must contain contributions only from GSB and PA. To isolate the GSB and PA signal contributions, the procedure described in Section S4 and S5 of the ESI† was followed. Briefly, we assumed that the PA band is independent of excitation wavelength and given approximately by the TA spectrum measured with 265 nm excitation at the same delay time. This PA-only signal was subtracted after scaling from the signal measured with visible excitation to yield the time-resolved GSB-only signals shown in Fig. 4 for perpendicular polarizations. The GSB signals obtained from spectra recorded with magic angle polarizations are similar (Fig. S5†), except that scattered pump light prevented measurements close to the excitation wavelength. The GSB signals in Fig. 4 reveal nearly symmetric bleaching on the red and blue sides of the excitation wavelength, supporting our neglect of SE. Simpson *et al.*^23^ also concluded that SE does not contribute to their negative Δ*A* signals from *Sepia* melanin.

The persistence of the hole beyond the time when pump and probe pulses are temporally overlapped (*i.e.*, *t* ≥ 500 fs) indicates that population holes are created and that the bleaching is not due to a coherent artifact.^29^ The hole width defined as the full-width at half-maximum (FWHM) of the best-fit Gaussian (Table S1†), is the same within experimental uncertainty in all measurements with the exception of the slightly larger hole width determined with excitation at 500 nm using perpendicular polarizations. The average hole width from all measurements is approximately 0.6 eV (5000 cm^−1^).

The observation of excitation wavelength-dependent spectral hole burning indicates unambiguously that the pump pulse only excites eumelanin chromophores that lie nearby in energy. If the very broad absorption spectrum of DOPA melanin (Fig. 1a) were due to a single kind of absorber, then the GSB signal would be the same for each excitation wavelength and would resemble the ground state absorption spectrum in Fig. 1a. Instead, the signals in Fig. 2 and 3 show that GSB is restricted to a band centered about the pump wavelength, which although wide (∼0.6 eV), is nonetheless much narrower than the absorption spectrum of DOPA melanin. These results, providing evidence of multiple kinds of chromophores in these absorption measurements, complement wavelength-dependent emission experiments that provided evidence of diverse absorbers with different HOMO–LUMO gaps.^9,10^

The excitation-dependent bleaching seen in Fig. 3 and 4 clarifies the GSB behavior of eumelanin reported at just a few pump and probe wavelengths in earlier ultrafast TA experiments.^2,19,20,22–24^ In measurements on natural *Sepia* melanin in aqueous solution, Nofsinger, Ye, and Simon reported negative TA signals with same-wavelength excitation and probing at 320, 350, and 380 nm.^2^ Warren and co-workers observed negative TA signals for several combinations of NIR pump and probe wavelengths.^20,22,23^

Our results also explain past reports of oppositely signed signals at the same probe wavelength.^21,24^ Aloi *et al.*^24^ reported a negative TA signal at a probe wavelength of 504 nm when exciting synthetic eumelanin in DMSO–methanol solution at 550 nm, yet Ye and Simon^21^ saw positive signals at this same probe wavelength when exciting at 303 nm. These observations are only apparently contradictory because spectral hole burning is only seen when the pump and probe wavelengths are sufficiently close as pointed out in pioneering work by Warren and co-workers.^19,20,22,23^ These authors showed using NIR TA measurements that negative, GSB-dominated signals seen for probe wavelengths sufficiently near the excitation wavelength, cross zero and become positive due to PA at wavelengths further removed from the pump wavelength.^23^ Our >2 eV (20 000 cm^−1^) probe window is approximately 20-fold wider than the one used in ref. 23, allowing us to capture the full hole profile on both the low and high energy sides of the excitation wavelength, and reveal how transient spectral hole burning depends on excitation wavelength.

The spectral holes in Fig. 4 do not change shape or center wavelength and simply decay to the baseline. A single inverted Gaussian function satisfactorily describes the bleach signal at all delay times (black curves in Fig. 4), although minor deviations are seen in the wings that may be due to assumptions made in our modeling procedure above. These broadband measurements of spectral holes in DOPA melanin show that the full hole profiles are actually Gaussian in shape, confirming a postulate by the authors of ref. 19.

### Transient spectral hole burning in systems with chemical disorder

Spectral hole burning is possible when the absorbers in a system can be differentiated by their transition energies over long enough time scales to be detected in a given experiment. The excitation pulse in a transient spectral hole burning experiment prepares a subensemble known as an ‘isochromat’,^30^ containing all molecules that absorb at the pump wavelength. Selective bleaching by this subensemble can burn a hole that is narrower than the full absorption spectrum, if exchange among the different absorbers or other line broadening mechanisms are minimal. For example, spectral holes can be burnt into the absorption band of a single type of absorber in a crystalline or glassy host when the inhomogeneous broadening arising typically from a distribution of site energies is greater than the homogeneous broadening experienced by the ensemble of absorbing molecules.^31^

Although spectral hole burning spectroscopy has been used almost exclusively to extract homogeneous line widths from an inhomogeneously broadened spectrum due to a single type of absorber, it does not matter whether all absorbers have the same chemical structure or whether a mixture of many types of absorbers is present. For a sample containing just one kind of chromophore, hole burning is generally only possible at cryogenic temperatures because the pure dephasing contribution to the homogeneous line width of a given absorber increases very rapidly with temperature.^32^ For this reason, hole burning is extremely difficult to observe for a single kind of absorber such as a dye molecule in room-temperature solution.^29^ In contrast, spectral hole burning can be much easier to detect in systems like eumelanin, which contain multiple kinds of chromophores, even at room temperature.

The transient spectral hole burning seen in our broadband TA measurements indicates that various chromophores of DOPA melanin have transition energies that are spread throughout its overall absorption spectrum. Each chromophore has an absorption line shape that reflects all of the line broadening mechanisms (inhomogeneity, lifetime broadening, pure dephasing, *etc.*) that are present. The observed hole is not due to transient bleaching of a single chemical species, but has contributions from all chromophores that can be excited by the pump pulse. In our experiment, the precise line shapes of the chromophores or how they are distributed throughout the band cannot be determined. However, the fact that the hole width in Fig. 4 and S5† is approximately independent of excitation wavelength suggests that the line shapes of individual chromophores are similar across the visible spectrum. Given this observation along with the Gaussian shape of the hole profile, we postulate simply that the line shape of every type of chromophore is approximately Gaussian with identical width (see details in the ESI Section S.4†). In this simple model, the line width (FWHM) of each absorber is given by the measured hole width of 0.6 eV divided by 
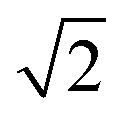
, or 0.4 eV (3000 cm^−1^). Such a width is typical for π → π* transitions of aromatic compounds in solution.

Transient bleaching in Fig. 4 is restricted chiefly to a symmetric Gaussian band centered about the excitation wavelength, although some deviations are noted in the tails. Generally, an organic molecule that absorbs at visible wavelengths exhibits additional allowed transitions in the UV that have considerable oscillator strength. For example, the indole quinone form of DHI is calculated to have much stronger absorption in the UV than in the visible.^33^ We note that Chen *et al.*^8^ were able to computationally reproduce the broad absorption spectrum of eumelanin by considering excitonic interactions among noncovalent assemblies containing only a single kind of chromophore, but with multiple electronic transitions. Given that ground state depletion due to photoexcitation should bleach transitions to all states reached from the ground state, why, for example, is hole burning at visible wavelengths not accompanied by hole burning at shorter wavelengths?

One possibility is that excitonic effects among chromophores enhance long-wavelength absorption while suppressing absorption at shorter wavelengths, focusing the oscillator strength in a single band. However, Chen *et al.*^8^ argued that due to geometric and packing disorder, any excitonic states should be scattered throughout the range of possible transition energies. An alternative explanation is that the interactions or environmental effects that cause different types of chromophores to absorb near the excitation wavelength may not cause them to have transitions in common at other wavelengths. Hole burning selects chromophores that absorb at a given wavelength, but other transitions for the absorbers in the isochromat are likely to vary widely in their transition energies and appear on the low- and high-energy sides of the hole, giving a broad background that would go undetected in our scaling/subtraction procedure. To better answer the question posed in the preceding paragraph, bleaching experiments should be observed over a wider spectral range, including in the UV.

### Transient bleaching dynamics provide insight into the role of couplings among chromophores

**Fig. 5:**
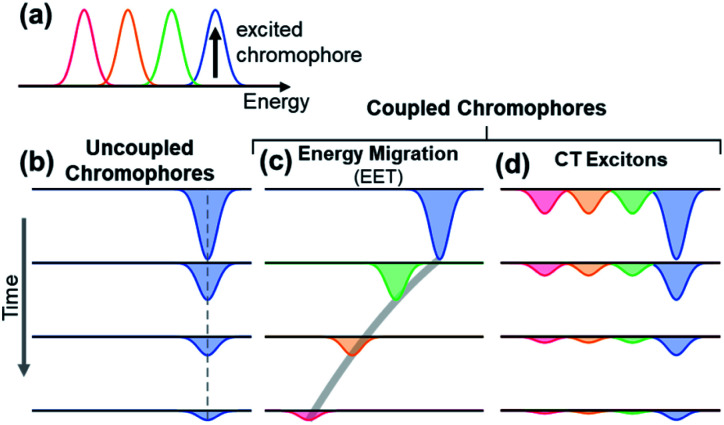
Simple illustrations of ground state bleach dynamics arising from possible excited state decay pathways in DOPA melanin. Examples are shown for 4 discrete chromophores that absorb at different energies as shown in (a). For illustration, the chromophore with the highest energy transition is excited (black vertical arrow). In uncoupled chromophores (b), only the excited chromophore is bleached; the bleach recovers over time due to excited state relaxation. In a system of coupled chromophores (c and d), additional chromophores can be bleached. (c) Energy migration by excitation energy transfer (EET) causes the bleach to redshift over time as energy is funneled from the excited chromophore to lower energy chromophores during excited state relaxation. (d) Charge transfer (CT) from the excited chromophore to neighboring chromophores forms charge transfer excitons that cause bleaching of all chromophores. The bleaching is strongest by the initially excited chromophore. Charge recombination restores the ground states, causing the bleach signals of the excited chromophore and its neighbors to decay in unison.

The observation of spectral hole burning near time zero provides clear evidence of chromophore heterogeneity in melanin (Fig. 4), but the subsequent hole filling dynamics provide even richer information about the role of inter-chromophoric couplings. Several possibilities for hole filling dynamics are illustrated in Fig. 5 for a system of a small number of different absorbers. The spectra of the chromophores are drawn as non-overlapping Gaussians of equal spectral width for simplicity (Fig. 5a). The black arrow in Fig. 5a represents photoexcitation by a pump pulse with a bandwidth that is narrow compared to the linewidth of the absorber.

As noted above, spectral hole burning is possible in a system containing many types of chromophores when absorption by a subset differs from the full absorption spectrum. A key issue is whether an initially excited chromophore can bleach a different one at a later time prior to excited state deactivation. This is not possible when the chromophores are distant from one another and non-interacting as in a dilute solution. In this case, additional kinds of chromophores beyond those initially present in the isochromat cannot be bleached at a later time, and the hole burned by the excitation pulse is stationary in frequency as hole filling occurs (Fig. 5b).

An excited state on one chromophore can cause bleaching of a second one if the two chromophores can interconvert or react. Two important cases are EET (Fig. 5c) and CT (Fig. 5d) between chromophores. EET is often observed in conjugated polymers, where it gives rise to energy migration by the incoherent diffusion of energy among chromophores.^34,35^ In this process, the excited state hops within a heterogeneous distribution of states, created by random breaks in electronic conjugation in the polymer backbone, and funnels to lower energy states. Energy migration by EET would produce a dynamic red shift in the spectral hole position with time as shown in Fig. 5c. On the other hand, CT between two chromophores causes bleaching of both the initially excited molecule and a second one. When a photoexcited chromophore can undergo CT with many different kinds of chromophores with roughly equal probabilities, then the bleaching of the absorption bands of the latter is attenuated statistically as shown in Fig. 5d.

The absence of any spectral evolution in the ground state bleach peaks in Fig. 4 argues against EET to eumelanin chromophores with lower transition energies. The dynamic red shifting that is expected to accompany energy migration is not seen with any of the visible excitation wavelengths (Fig. 4). These results suggest that the chromophores in melanin and their spatial organization do not permit the transfer or funneling of excitation energy, a conclusion supported by the wavelength-dependent emission seen in eumelanin.^9,10^

**Fig. 6:**
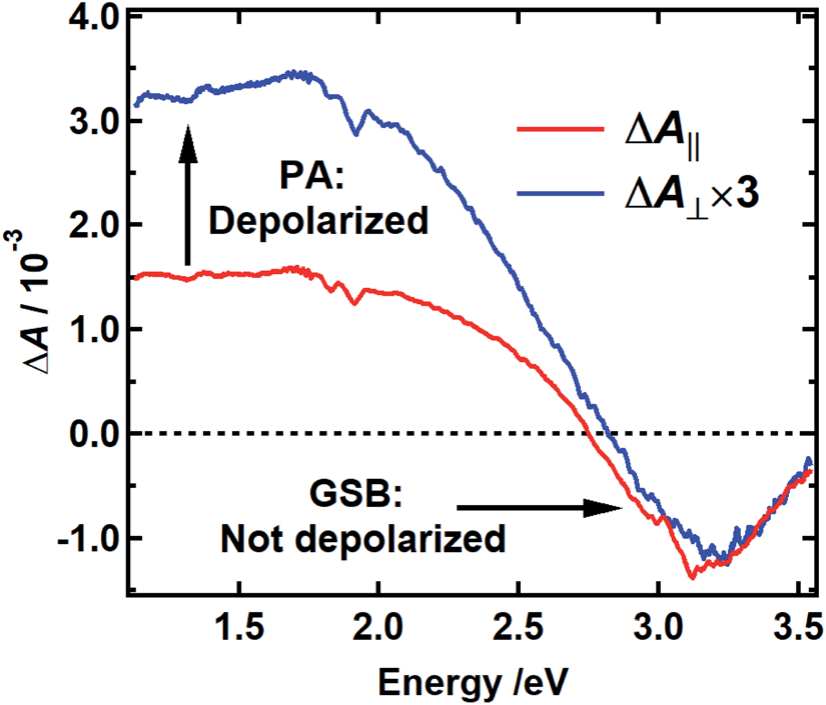
Polarization-dependent transient absorption spectrum of DOPA melanin with 400 nm excitation recorded at 250 fs time delay with perpendicular (blue) and parallel pump–probe polarizations (red). The spectrum recorded with perpendicular polarizations is scaled by a factor of 3. The small feature around 3.1 eV in the red curve is an artifact from pump scatter.

To investigate the possibility that EET takes place only among chromophores with similar transition energies, a possibility that would not be detectable as a dynamic red shift, we examined the initial anisotropy of the DOPA melanin TA spectra recorded with parallel and perpendicular pump and probe polarizations (Fig. 6). The anisotropy of a TA signal decays when the transition dipole moment (TDM) direction of the probed transition changes with time due to reorientation of a chromophore in the lab frame or due to energy transfer to a second chromophore with a different orientation.^36^ For example, rapid energy transfer in stacks of perylene molecules leads to a rapid decay of the anisotropy in ∼1 ps.^37^

The spectra recorded with parallel and perpendicular polarizations 250 fs after excitation at 400 nm are shown by the red and blue curves in Fig. 6, respectively. The TA spectra for parallel and perpendicular polarizations at different times are presented in Fig. S6 and S2c† respectively. This pump wavelength was chosen because the PA signal, which is assumed to be equal to the TA spectrum measured with excitation at 265 nm, is near zero at a probe wavelength of 390 nm (3.2 eV), where the most negative TA signals are seen (Fig. 3b and S2c†). In this case, the signal at 390 nm measures the anisotropy associated with GSB. The signal at 800 nm (1.5 eV), a wavelength distant from the pump wavelength where no GSB is expected, allows the anisotropy associated with PA to be monitored.

Anisotropy theory predicts that the signal recorded using perpendicular pump and probe polarizations will be three times lower in intensity than one recorded using parallel polarizations when there is no depolarization of the bleached chromophores.^38^ The GSB signal in Fig. 6 near 3.2 eV shows that the TDM directions of the bleached chromophores are unchanged 250 fs after excitation because the blue and red traces are nearly coincident. The high degree of polarization suggests that the initially excited chromophore does not reorient. Because the initial anisotropy of the pure bleach signal is not lost 250 fs after excitation, and because the anisotropy remains unchanged for the remainder of the decay (Fig. S7†), we rule out depolarization due to energy transfer between chromophores with similar transition energies. In contrast, the perpendicular signal multiplied by 3 is much larger than the parallel signal at probe wavelengths where the PA signal is dominant (black vertical arrow in Fig. 6). This indicates that the transient state probed in the NIR rapidly loses memory of the initial pump polarization. One explanation for this behavior is the creation of an immobile CT state that forms either intramolecularly or as a result of charge transfer between chromophores.^39,40^ In an immobile CT state the bleached chromophore does not reorient, and instead the probed transition dipole moment of the CT state either makes a much different angle relative to the pump polarization or is randomized over a range of angles due to orientational disorder of the charge acceptor. Note that an alternative state, such as a singlet excited state of an uncoupled chromophore, could also possess a transition dipole moment at a much different relative angle.

### Eumelanin's heterogeneous chromophores have common PA spectra and decay kinetics

While the above evidence rules out interchromophoric EET in DOPA melanin, the observed hole filling dynamics could arise from either noninteracting chromophores (Fig. 5b) or inter-chromophore CT (Fig. 5d). We examine the spectra and decay kinetics measured for the PA band with different excitation wavelengths to decide between these possibilities.

**Fig. 7:**
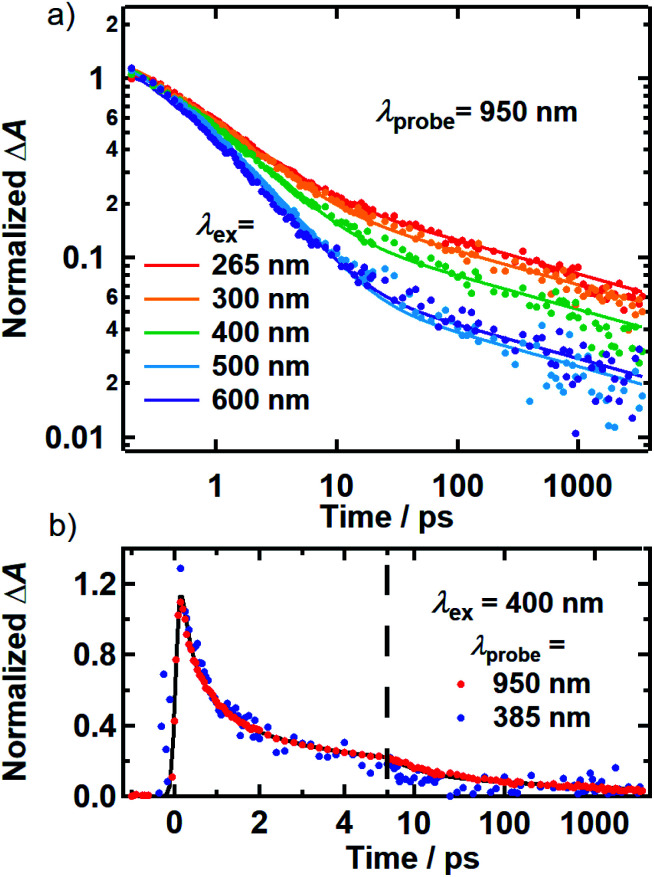
(a) Kinetics probed at 950 nm for 265 (red), 300 (orange), 400 (green), 500 (blue) and 600 nm (purple) excitation. Solid lines are best-fit curves of the model function discussed in the text. The kinetic traces shown were measured using the magic angle pump–probe polarization condition. All kinetics were normalized to be unity at 250 fs. (b) Kinetics for 400 nm excitation at probe wavelengths of 900 and 385 nm.

The normalized decays of the PA band at 950 nm measured at each excitation wavelength are complex and cannot be fit to a single exponential function (Fig. 7a). Whereas the authors of previous TA studies fit their kinetic traces to 2 or 3 exponentials,^21,25,41^ a log–log plot (Fig. 7b) reveals that the kinetics are actually biphasic in nature, and better fits are obtained with a similar number of parameters using a linear combination of a stretched exponential and a power law,1
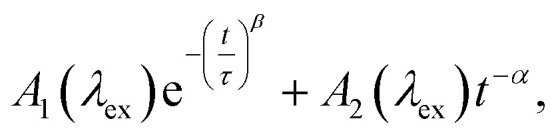
where *A*_1_ and *A*_2_ are amplitudes that depend on the excitation wavelength, *λ*_ex_, *β* is the stretching parameter for the stretched exponential with lifetime *τ*, and *α* is the exponent of the power law term. During fitting, all parameters were constrained except for the normalization and weighting parameters (see ESI† Section S7 for detailed discussion). This indicates that irrespective of the excitation wavelength, the PA band decays with similar rates, albeit with varying proportions of the stretched exponential and power law contributions. Further details and interpretations of the decay signals will be the focus of a forthcoming report.

The decay kinetics of the positive and negative TA signals probed at 950 nm and 385 nm, respectively, are compared in Fig. 7b for 400 nm excitation. As seen in Fig. 3b, the GSB and PA bands overlap minimally at these probe wavelengths, directly revealing their decay kinetics. The ground state recovery kinetics at 385 nm (blue markers) are identical within experimental uncertainty to the decay kinetics of the PA band at 950 nm (red markers) after the former signal is inverted and normalized to unity. Decomposing the TA spectra recorded using 500 and 600 nm excitation yields similar results, whereby the GSB recovery kinetics match the PA decay kinetics (Fig. S9†). The matching kinetic signals indicate that the transient population observed in our TA measurements decays to restore the ground state. This agreement also means that 80–95% of the light energy is dissipated in the first 10 ps in DOPA melanin for all of the studied excitation wavelengths.

Together, the transient spectra and kinetics (Fig. 2, 3 and 7) reveal a surprisingly homogeneous response by DOPA melanin to photoexcitation, even when the absorbed photon energy is tuned by more than a factor of two from the UV to the visible. Whereas others have reported multiexponential decay kinetics that vary with pump and probe wavelengths,^21,24,25,42^ our measurements reveal unexpectedly similar excited state deactivation, offering an explanation of how eumelanin is able to effectively dissipate light with wavelengths throughout its broad absorption spectrum. The spectral hole burning reported here provides evidence for the selective excitation of subensembles of chromophores. If these chromophores were non-interacting, then each subensemble should give rise to its own characteristic transient spectra and dynamics based on the very reasonable assumption that the chromophores are just as differentiated by their excited state properties as by their ground-state absorption. The observation of a nearly identical response from each subensemble is thus a strong argument for significant excited-state interactions among the DOPA melanin chromophores.

A key issue is whether the similar PA signals are due to absorption by a single species formed regardless of which group of chromophores is excited, or whether it arises from many different transient species, created in approximately the same amounts from every subensemble. In support of the first possibility, spectrally broad TA in the NIR is a hallmark for weakly bound electrons. It is possible that photoexcitation creates an electron hole pair in which an electron or hole is captured faster than our time resolution by a trap site on the periphery of a melanin protomolecule (see Fig. 1d). The decay of the signal would then be due to charge recombination. However, it is difficult to explain why recombination would occur with identical rates, no matter what kind of chromophore acted as the electron or hole donor.

For this reason, we favor the second possibility, in which the common PA band and dynamics represent an average signal from a diverse group of states. The broad PA spectrum of DOPA melanin is essentially as broad and structureless as the ground state absorption spectrum, suggesting that it could be due to a superposition of narrower PA bands from many separate chromophores, provided that the same set of PA-producing states is formed by all excitation wavelengths. CT interactions among chromophores can explain this behavior, if electron transfer between one kind of chromophore and any other is equally probable. For example, if a hole that is localized on the chromophore selected by the pump laser absorbs weakly or not at all, then the PA signal will be dominated by absorption from the many different kinds of electron acceptors, which are approximately the same for any excitation wavelength.

Although additional study is needed, this behavior could be explained by the ultrafast formation of an immobile charge transfer (CT) state that forms intramolecularly, or as a result of CT between different chromophores.^40,43^ Electron–hole recombination among diverse donor–acceptor pairs would be expected to occur with different rates consistent with the observation of stretched exponential decays. The unchanging anisotropy in the GSB signal suggests that the hole or electron remains immobile and localized on the chromophore that was initially excited. On the other hand, the low anisotropy of the PA band is consistent with electron or hole transfer from each of the initially photoexcited chromophores to a second chromophore. For example, the PA band could correspond to a CT transition with a transition dipole moment oriented much differently (*e.g.*, out of plane) than the photoexcited chromophore (in-plane).^40^

We suggest that CT exciton formation in <200 fs best explains the TA dynamics of DOPA melanin (Fig. 5d). CT excitons could be formed either within a protomolecule or between layered protomolecules. It is difficult to decide among these possibilities on the basis of the present measurements, but we note that stacked layers and π–π interactions among aromatic chromophores are a ubiquitous motif in carbon nanomaterials, including eumelanin (see below). Close interlayer separation can lead to the facile formation of CT excitons in systems of π-stacked and excitonically coupled chromophores.^44^ In fact, CT excitons can form within the time resolution of ultrafast laser experiments (<10 fs) as seen, for example, in conjugated polymers.^45,46^ Therefore, we propose that efficient recombination of a CT state on the few ps time scale is the principal event behind the photostability of eumelanin.

### Raman spectra suggest that variation in the size of sp^2^-hybridized domains contributes to chromophore heterogeneity

The transient spectral hole burning experiments reveal that the electronic absorption spectrum of DOPA melanin is due to many chromophores with distributed transition energies, but the causes of this heterogeneity are still uncertain. Several computational studies of putative melanin chromophores show that larger, more conjugated chromophores absorb at longer wavelengths,^8,33,47^ but experimental evidence is lacking. Raman spectroscopy has been shown to be a sensitive probe of conjugation length in organic materials. For example, a Raman shift of the C=C stretching peaks is seen in polyenes depending on their number of alternating double bonds.^48^ In a heterogeneous material, as the laser wavelength comes into resonance with chromophores that differ in the size of their conjugated regions, their Raman signal becomes resonantly enhanced, producing characteristic shifts (‘dispersion’) of the observed Raman bands.^49^ For this reason, Raman spectroscopy has been shown to be a sensitive probe of the sp^2^-hybridized content in disordered carbon materials in which dispersion–size relationships are well-established.^50^ Inspired by a study noting similarities between the Raman spectra of natural melanins and disordered carbon materials,^51^ we apply insights from studies of disordered carbon materials^50^ for the first time to learn about the size and sp^2^-hybridized content of the chromophores in DOPA melanin.

**Fig. 8:**
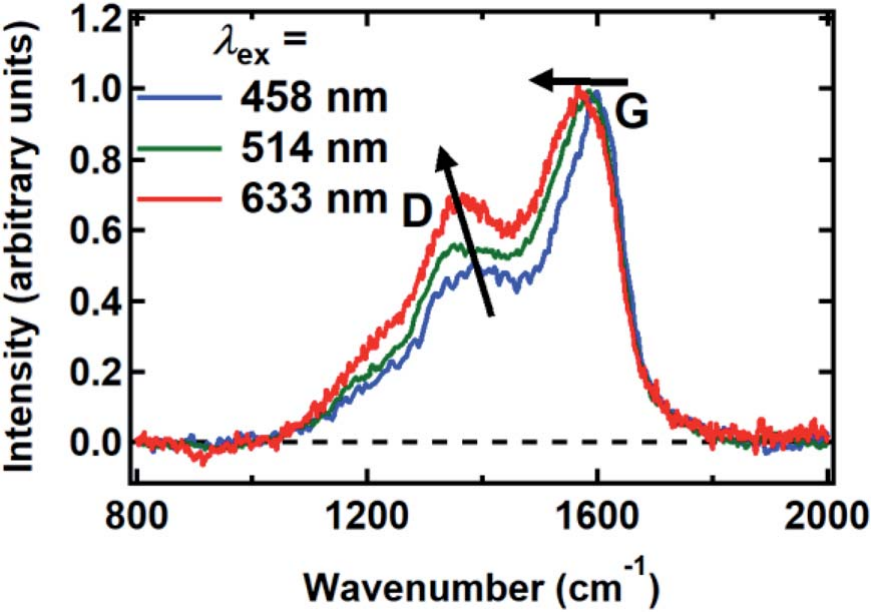
Raman spectra of DOPA melanin measured with the laser wavelengths indicated. The changes in the G and D bands with increasing wavelength are indicated by arrows. The spectra were normalized to have a peak G band intensity of unity.

Raman spectra were recorded between 800 and 2000 cm^−1^ from spin-coated DOPA melanin thin films using multiple laser wavelengths (Fig. 8). The spectra feature two prominent bands, centered at 1365 cm^−1^ and 1585 cm^−1^ for excitation at 514 nm, that closely resemble the “D” (“disorder”) and the “G” (“graphitic”) bands seen in the Raman spectra of disordered carbon materials.^50^ In these materials, the G band originates from C=C stretching in sp^2^-hybridized domains, while the D band is due to breathing modes in six-membered rings of sp^2^ carbon atoms.^50^ These bands are consistent with the presence of disordered carbonaceous nanostructures in DOPA melanin.

Both the D and G bands measured for DOPA melanin (Fig. 8) exhibit dispersion, shifting to lower frequency as the wavelength of the excitation laser is increased. The D and G bands in Fig. 8 also exhibit an increase in their intensity ratio as the laser wavelength is tuned from 458 nm to 633 nm. These trends, which are typical in disordered carbonaceous materials,^50,52,53^ signal that there is a distribution of sp^2^-hybridized domains of different size in DOPA melanin. Altogether, these results support the conclusion that the spectral hole burning seen in Fig. 3 and 4 originates from exciting chromophores differentiated by the size of their sp^2^ domains, although we cannot rule out additional effects due to redox state or packing disorder.

Ferrari and Robertson have extensively correlated changes in the D and G peaks in diverse carbon materials that range from graphite to amorphous diamond.^49,50,54^ Additionally, Ferrari and Robertson showed that the Raman spectra of carbon nitrides and carbon-only films are extremely similar.^54^ Therefore, the D and G peak correlations are still applicable to synthetic and natural melanins despite having a carbon-to-nitrogen mole ratio of approximately 8.^55^

Additional size information can be inferred from the Raman spectrum of DOPA melanin using the phenomenological model developed by Ferrari and Robertson for various phases of disordered carbon (see Section S9 in the ESI† for details).^50^ Based on the behavior of the D and G bands discussed above, the Raman spectra of DOPA melanin appear intermediate between those of nanocrystalline graphitic carbon and amorphous carbon. In this range, the D:G peak height ratio scales with the square of the sp^2^ domain size.^50^ Using this relationship, we find that the chromophores in DOPA melanin, which are resonant with 514 nm excitation, have an sp^2^ domain length of ∼1 nm (see Section S9 in the ESI†). Although crudely determined, this length is consistent with the size of planar protomolecules made from a small number (*e.g.*, 4–8) of DHI or DHICA units (for an example structure, see Fig. 1d).^56,57^

### Melanin and disordered carbon nanomaterials have common excited state dynamics

Meredith and co-workers first noted in an insightful paper a decade ago that eumelanin and disordered carbon materials like carbon black have virtually identical steady-state absorption spectra and similar graphitic structures as seen in TEM images.^58^ Earlier, Cheng *et al.*^59^ concluded from their X-ray diffraction measurements that synthetic tyrosine eumelanin consists of planar sheets made from 4–8 DHI units that are stacked in graphite-like layers. This structure, described as a ‘paracrystalline array’ in ref. 59, is strongly reminiscent of structures currently discussed for carbon nanodots,^60^ which also have absorption spectra very similar to those of melanins.

**Fig. 9:**
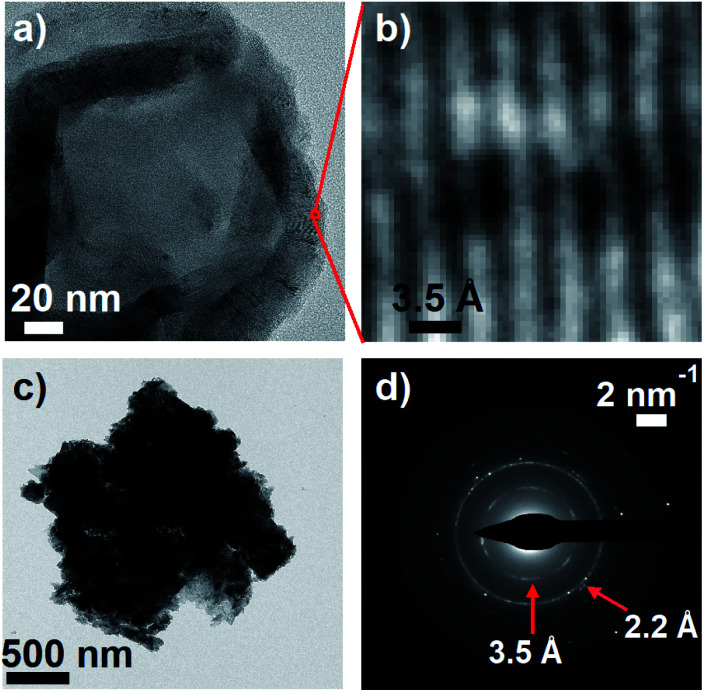
(a) Transmission electron microscopy (TEM) image of DOPA melanin showing graphite-like stacked layers in the dried material. (b) An expanded view of the indicated region in panel a, showing diffraction. (c) A dense mass of DOPA melanin. (d) The selected area electron diffraction (SAED) pattern from the entire mass in panel c.

To further explore parallels between eumelanin and carbon nanomaterials, transmission electron microscopy (TEM) images of DOPA melanin that was drop-cast onto carbon supports were recorded. The TEM images show clear diffraction contrast (Fig. 9a and b) with a spacing of 3.5 Å that matches the distance between stacked layers in graphitic carbon nanomaterials. The value of 3.5 Å agrees well with the value of 3.45 Å determined in X-ray scattering measurements on eumelanin synthesized from tyrosine,^59^ and is somewhat smaller than the inter-sheet distance of between 3.7 and 4.0 Å determined by Watt *et al.*^58^

A selected area electron diffraction (SAED) measurement on a DOPA melanin agglomerate shown in Fig. 9c reveals an electron diffraction pattern with characteristics typical of graphitic sheet-like structures (Fig. 9d). The diffraction pattern exhibits a 2.2 Å spacing, which we assign to in-plane periodicity based on the observation of similar spacings in graphene quantum dots and graphite.^61,62^

These TEM results and the Raman spectra discussed in the previous section add to the similarities Meredith and co-workers noted between static properties of melanin and graphitic carbon materials like carbon black.^58^ However, our observations of transient hole burning, broad PA at NIR wavelengths, and complex, biphasic decay kinetics in photoexcited DOPA melanin closely parallel similar observations from TA studies on a range of disordered carbon nanomaterials, including carbon dots, graphene oxide, and graphene quantum dots.^63–67^ For example, Wang *et al.*^63^ tuned the excitation wavelength in their femtosecond TA experiments across the absorption spectrum of graphene oxide and observed a NIR PA band in addition to excitation wavelength-dependent bleaching or hole burning, consistent with the existence of discrete chromophores. Fig. S10 in the ESI† shows just how similar our TA spectra of DOPA melanin are to the graphene oxide results presented in ref. 63.

Although the chemical structures of the actual chromophores or protomolecules almost certainly show a great deal of variation for different carbon nanomaterials, they are all thought to have chemically heterogeneous domains or islands of sp^2^-hybridized carbon and nitrogen atoms, which define the chromophores and are likely to interact with one another in similar ways. Disorder and these common organizational motifs are likely responsible for their fascinating photophysical properties. The results of this study demonstrate the power of excitation wavelength-dependent ultrabroadband TA spectroscopy for disentangling the excited state dynamics in disordered systems with extreme chromophore heterogeneity.

We note that our estimated absorption line width (0.4 eV) for eumelanin's visible chromophores is similar to the widths observed in UV-Vis absorption spectra of graphene-like chromophores with well-defined structures.^68,69^ Our estimated width is ∼50% greater than the 0.28 eV line width (FWHM) determined in recent transient spectral hole burning experiments on graphitic carbon nitride quantum dots.^64^ However, this difference could be due to a greater degree of disorder in DOPA melanin than in the carbon nanodots.

These parallels in structure and dynamics strengthen the connections between the ubiquitous eumelanin pigments of biology and the carbonaceous nanomaterials of materials science. From the standpoint of photophysics, eumelanin appears to be highly similar to carbon nanodots with very low fluorescence quantum yields. We propose that eumelanin should be viewed in the same category as disordered carbon-rich nanomaterials such as graphene oxides, graphitic carbon nitrides, and other carbon or graphene dots. Based on the common dynamics seen across this class of materials, insights from one system will almost certainly advance understanding of structure–function relations in others. The fact that melanin is synthesized enzymatically under physiological conditions also offers the tantalizing possibility of low-temperature routes for synthesizing novel carbon materials with unusual, tailorable, and biocompatible photonic properties.^58^

## Conclusions and outlook

The broadband femtosecond TA spectra reported in this study reveal transient hole burning in DOPA melanin, demonstrating that photoexcitation selectively interrogates different subsets of its chemically heterogeneous chromophores and providing the first account of their intrinsic spectroscopic line shapes and dynamics. We have shown that the excited state dynamics of each chromophore subset can be tracked in the presence of full environmental heterogeneity in eumelanin, providing insights into the actual eumelanin polymer that cannot be obtained from studies of small molecule subunits. This study, and a recent work by Sciortino *et al.*^64^ on the photophysics of carbon nanodots, encourage the application of transient hole burning spectroscopy, which is typically used to study a single chromophore in heterogeneous environments, for disentangling the excited state dynamics in disordered systems made up of many kinds of chromophores, like in carbonaceous nanomaterials.

The constancy of the PA band measured using excitation wavelengths from 265–600 nm, and its common decay kinetics, show that the chromophores present in eumelanin undergo a common electronic deactivation mechanism regardless of their size or precise structure. The similar kinetic decay profiles and deactivation times reflect an element of disorder that is common to all subsets of excited chromophores present in eumelanin. The spectroscopic and TEM observations in this study implicate CT interactions among two-dimensional chromophores. Our results offer a new perspective on the photodynamics of eumelanin, suggesting that excitations initiate ultrafast charge transfer among sp^2^-hybridized domains, which are organized as in graphitic carbon materials. However, future work is needed to better define the heterogeneous chromophores of eumelanin and their spatial organization.

Spectroscopic, as well as TEM imaging experiments, reveal that melanin has much in common with carbonaceous nanomaterials. While these parallels in steady-state properties have been touched upon briefly in the past,^58^ and while it was suggested that the self-assembly of graphene fragments and eumelanin are likely to be similar,^70^ this study extends these parallels for the first time to excited state dynamics. Future work is needed to understand what the common photophysics in these carbonaceous materials imply about the size of conjugated domains in their chromophores and structural motifs such as π–π stacking. Understanding the factors that influence the line shape and electronic line broadening of the chromophores of eumelanin will be invaluable not just for understanding this biological pigment, but also for developing structure–function models for broad classes of carbonaceous nanomaterials.

## Conflicts of interest

There are no conflicts to declare.

## Supplementary Material

SC-011-C9SC04527A-s001

SC-011-C9SC04527A-s002
