# Supplementary material for: Ultrafast spectral hole burning reveals the distinct chromophores in eumelanin and their common photoresponse
Source: Chem Sci. 2019 Dec 18;11(5):1248–59. doi: 10.1039/c9sc04527a (PMC8148383; doi:10.1039/c9sc04527a)
Supplement: SC-011-C9SC04527A-s001 [file SC-011-C9SC04527A-s001.pdf]

Electronic Supplementary Material (ESI) for Chemical Science.
This journal is © The Royal Society of Chemistry 2019

Electronic Supplementary Information for

Ultrafast spectral hole burning reveals the distinct chromophores in eumelanin and their common photoresponse

Forrest R. Kohl,† Christopher Grieco,† and Bern Kohler*

Department of Chemistry and Biochemistry, The Ohio State University, 100 West 18th Avenue, Columbus, Ohio 43210, United States

† F.R.K. and C.G. contributed equally to this work.

*Corresponding Author: Bern Kohler: kohler.40@osu.edu, Tel: +1 614-688-2635

Table of Contents

S.1. Methods 2

S.2. Normalized transient absorption spectrum of DOPA melanin. 4

S.3. Transient absorption spectra of DOPA melanin using all excitation wavelengths 5

S4. Model for separating ground state bleach and photoinduced absorption contributions to the melanin TA signals. 7

S.5. Procedure for subtracting positive contributions to the transient absorption signals in DOPA melanin measured using 400, 500, and 600 nm excitation 9

S.6. Time-dependent anisotropy for 400 nm excitation of DOPA melanin 11

S.7. Modeling parameters for transient absorption kinetics at 950 nm 12

S.8. Isolated ground state bleach kinetics using 500 and 600 nm excitation 14

S.9. Size estimation of DOPA melanin sp^2 domains from Raman bands 15

S.10. Spectral hole burning of DOPA melanin and graphene oxide 16

S.11. References 17

S.1. Methods

DOPA melanin synthesis and purification

DOPA melanin was synthesized as described in the literature.[1] All chemicals were used as received. Specifically, 1 g of L-DOPA (Sigma Aldrich, ≥ 98%) was dissolved in 200 mL of ultrapure water in a round bottom flask to yield a 25 mM solution. Aqueous ammonium hydroxide (28 – 30% by mass, Fisher Chemical) was then added to raise the pH to 9.5. Air was bubbled into the round bottom flask and the mixture was stirred gently. After three days, ~200 mL of acetonitrile (Sigma Aldrich, ≥ 99.5%) was added to the solution until the DOPA melanin flocculated. The contents of the flask were then centrifuged at 10,000 g to yield a solid pellet, and a tan colored supernatant. The supernatant was removed, fresh acetonitrile was added, and centrifugation was repeated. After 2-3 cycles of centrifugation, the supernatant was clear and colorless and lacked UV-visible absorption peaks above 250 nm. The final solid pellet was isolated and dried under nitrogen. The resulting shiny black flakes dissolve easily in water.

Steady-State UV-visible Spectroscopy

UV-visible absorption spectra were recorded at room temperature. The UV-visible spectrum of DOPA melanin in Figure 1a in the main text was recorded with a Cary 5000 UV-Vis-NIR spectrophotometer using a 1 mm path length fused silica cuvette. The sample concentration of the melanin solution was 0.14 mg mL^{-1}.

Transmission Electron Microscopy

TEM samples were prepared by drop casting aqueous DOPA melanin solutions onto planar carbon supports. An FEI Tecnai G2 30 was used for analysis of the dried DOPA melanin using and accelerating voltage of 200 kV.

Raman Spectroscopy

Raman spectroscopy was performed using a Renishaw inVia Raman microscope. The sample was prepared by spin-coating a DOPA melanin film onto a calcium fluoride substrate from an aqueous solution. The laser wavelengths used were 458, 514, and 633 nm. The spectrometer was calibrated using the 520 cm^{-1} Raman shift of a silicon reference for each wavelength. Multiple scans were performed and averaged at different positions on the film. No changes in the Raman spectrum were seen at various points on the film, confirming that film uniformity was achieved through the spin-coating method. The photoluminescence background, which was relatively weak due to the low emission yield of DOPA melanin, was removed from the spectra: A third order polynomial was fit to points in the spectra that were free from Raman scatter from the sample (i.e., < 900 cm^{-1} and > 2000 cm^{-1}). This polynomial fit was then subtracted from the data to yield the Raman spectra presented in the main text.

Broadband UV/visible/NIR transient absorption

Femtosecond broadband TA measurements were performed using a previously described spectrometer with separate detectors for UV-Vis and Vis-NIR broadband detection.[2, 3] At each pump wavelength, broadband transient absorption spectra were acquired with each detector in separate experiments using overlapping probing windows: 350 – 700 nm (UV-Vis) and 650 – 1150 nm (Vis-NIR). The spectra were then joined as described below. The pump beam was attenuated using a variable neutral density filter and focused to a spot size (defined as the $1/e^2$ Gaussian beam radius and measured

by the scanning knife-edge method) of ~1000 µm for Vis-NIR experiments and ~500 µm for UV-Vis experiments. The pump and probe pulses were linearly polarized, and a half wave plate was used to adjust the angle between the polarizations. Reported pump fluences represent on-axis (r = 0) values calculated assuming Gaussian pulses in space and time. The probe beam was focused to a spot size of 200 µm at the sample for both UV-Vis and Vis-NIR measurements. Approximately 4 mL of DOPA melanin solution was recirculated using a liquid flow cell, which consisted of a 1 mm thick PTFE spacer sandwiched between two CaF_2 windows of thickness 1 mm (front) and 2 mm (back).

To ensure that the pump beam energy density was approximately equal for UV-Vis and Vis-NIR TA measurements conducted at each pump wavelength, a gradient neutral density filter was used to attenuate the pump beam until the ΔA signal intensity of the DOPA melanin sample at 650 nm at a delay time of 500 fs was approximately 1 x 10^{-3}. Even with this step, experimental fluctuations caused the signals in the region of spectral overlap to differ slightly, so the transient spectra collected separately with the UV/Vis and Vis/NIR spectrometers were joined by first scaling the Vis/NIR spectrum to best match the UV/Vis spectrum in the region of overlap (650 – 700 nm) before averaging the two data sets. The scaling factor was always within 10% of unity.

Samples used in TA experiments were prepared by dissolving dried DOPA melanin, synthesized as described above, in pH 7 phosphate buffer solution prepared with water from an ultrapurifier to yield a melanin concentration of 0.29 mg mL^{-1}. Fresh 4 mL aliquots of a stock solution were used for acquiring data at each excitation wavelength. The absorbance of the solution in a 1 mm path length cell at the different pump wavelengths (in parentheses) were 0.11 (600 nm), 0.22 (500 nm), 0.45 (400 nm), 0.75 (300 nm), and 0.90 (265 nm). No changes in the UV-visible spectrum of the DOPA-melanin sample were observed after transient absorption experiments.

S.2. Normalized transient absorption spectrum of DOPA melanin

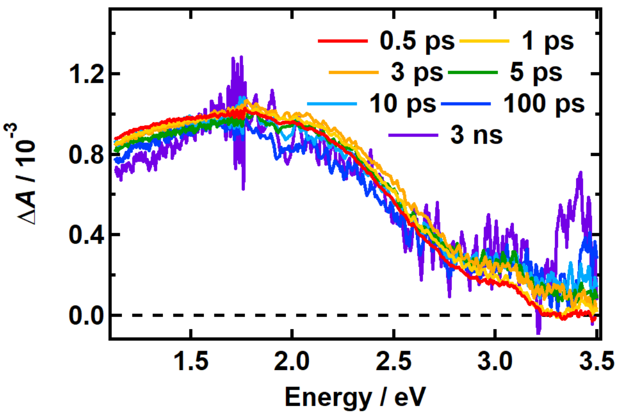

Figure S1. Normalized transient absorption spectra of DOPA melanin excited using 265 nm at the indicated delay times. The spectra were recorded using the magic angle pump-probe polarization condition.

S.3. Transient absorption spectra of DOPA melanin using all excitation wavelengths

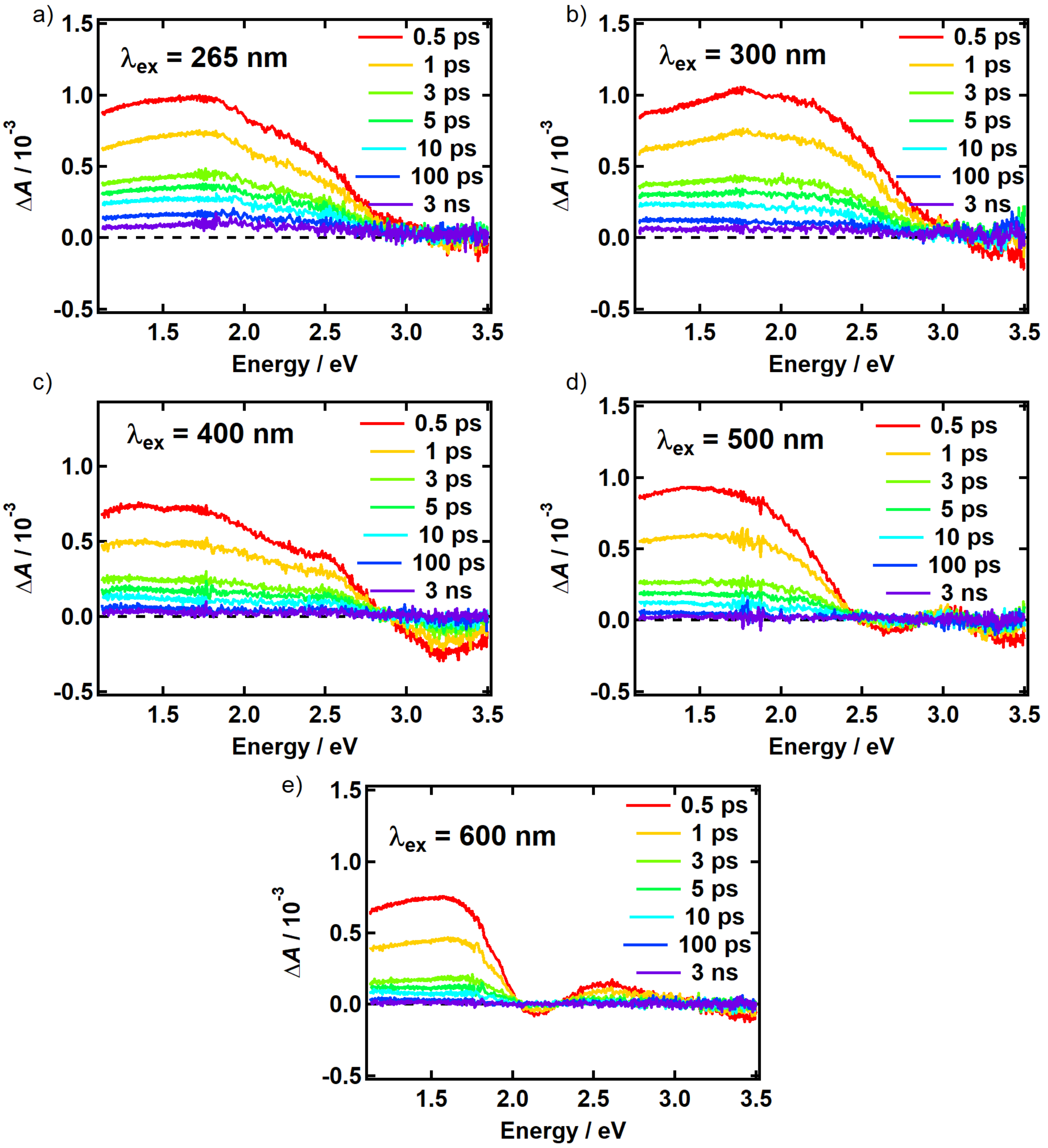

Figure S2. Transient absorption spectra of DOPA melanin excited at **a)** 265 nm, **b)**300 nm, **c)** 400 nm, **d)** 500 nm, and **e)** 600 nm and at the indicated delay times using perpendicular pump and probe polarizations.

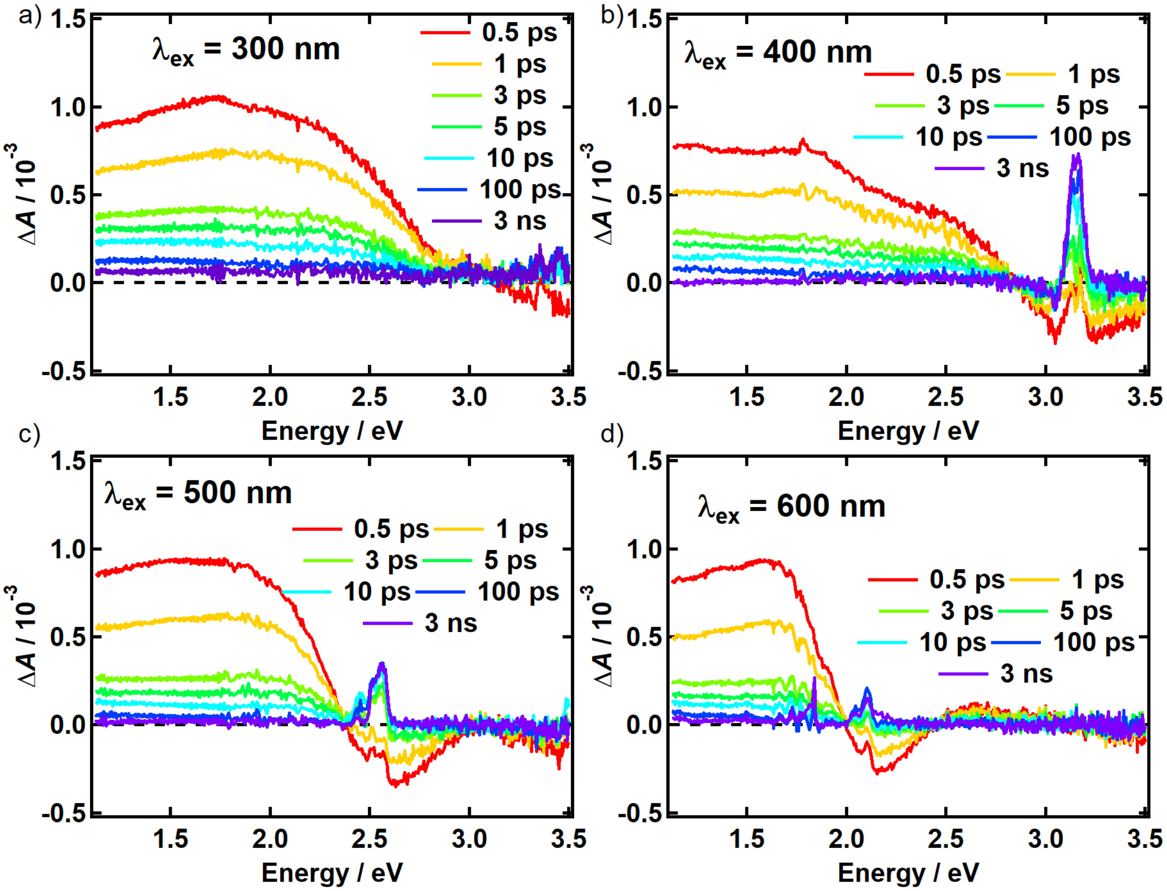

Figure S3. Transient absorption spectra of DOPA melanin excited using **a)** 300 nm **b)** 400 nm **c)** 500 nm and **d)** 600 nm excitation wavelengths at the indicated delay times using magic angle pump-probe polarization conditions.

S.4. Model for separating ground state bleach and photoinduced absorption contributions to the melanin TA signals

This section presents a simple model for separating ground state bleach from photoinduced absorption signal contributions in a system with many absorbers and applies it to melanin. The model nominally assumes that the absorbers behave independently and do not undergo bimolecular reactions such as excitation energy transfer and electron transfer. However, this model is approximately correct for a system with rapid electron transfer among chromophores as explained in the text.

Each absorber has a unique ground state spectrum given by cross section $\sigma_i(\nu)$, where ν is frequency and the subscript i indexes all the distinct absorbers in the system. We assume that excitation of the ith absorber at frequency ν populates a transient state with photoinduced absorption (PA) cross section $\sigma_i'(\nu)$. This state may be the excited state reached in the initial transition induced by frequency ν or it could be a different state populated from the first, much faster than can be experimentally resolved. We assume further that all absorption cross sections are independent of time.

Neglecting stimulated emission, the TA signal measured at pump and probe frequencies of ν_{pu} and ν_{pr}, respectively, can be decomposed into the sum of a PA contribution and a ground state bleach (GSB) contribution,

$$\Delta A(\nu_{\mathrm{pu}},\nu_{\mathrm{pr}}) = \Delta A^{\mathrm{GSB}}(\nu_{\mathrm{pu}},\nu_{\mathrm{pr}}) + \Delta A^{\mathrm{PA}}(\nu_{\mathrm{pu}},\nu_{\mathrm{pr}}). \tag{S1}$$

In a system of multiple absorbers, the total signal at delay time t between the pump and probe pulses can be written,[4]

$$\Delta A(\nu_{\mathrm{pu}},\nu_{\mathrm{pr}};t) \propto \sum_i N_i \sigma_i(\nu_{\mathrm{pu}})\left[\sigma_i'(\nu_{\mathrm{pr}}) - \sigma_i(\nu_{\mathrm{pr}})\right] R_i(t), \tag{S2}$$

where N_i is the number density (i.e., the number of absorbers per unit volume) of the ith absorber in its ground state, and the response function $R_i(t)$ describes the (initial) decay kinetics of the transient state with absorption cross section $\sigma_i'(\nu)$. Eq. S2 is valid if there are only two states (ground state and the photoexcited state) for each absorber. If additional states can be populated during relaxation, eq. S2 is still valid at times before these additional states are populated significantly. We assume further that the decay kinetics are approximately the same for all absorbers, consistent with the observations discussed in the main text. In that case, $R_i(t)$ is the same function, $R(t)$, for all absorbers, and can be dropped as we will assume that the delay time is the same for all quantities in the following equations.

The right-hand side of eq. S2 can be decomposed into PA and GSB contributions,

$$\Delta A^{\mathrm{PA}}(\nu_{\mathrm{pu}},\nu_{\mathrm{pr}}) \propto \sum_i N_i \sigma_i(\nu_{\mathrm{pu}}) \sigma_i'(\nu_{\mathrm{pr}}) \tag{S3}$$

$$\Delta A^{\mathrm{GSB}}(\nu_{pu},\nu_{\mathrm{pr}}) \propto -\sum_i N_i \sigma_i(\nu_{pu}) \sigma_i(\nu_{\mathrm{pr}}). \tag{S4}$$

Eq. S4 shows that contributions to the GSB signal in a system of non-interacting chromophores arise only from those absorbers for which the ground state absorption cross section is non-zero at both the pump and probe frequencies.

For the visible pump frequencies, $\nu_{\mathrm{pu}}^{\mathrm{vis}}$, used in our measurements, both PA and GSB signal contributions are present. Eq. S1 can be rearranged to solve for the GSB signal,

$$\Delta A^{\mathrm{GSB}}\left(\nu_{\mathrm{pu}}^{\mathrm{vis}},\nu_{\mathrm{pr}}\right)=\Delta A\left(\nu_{\mathrm{pu}}^{\mathrm{vis}},\nu_{\mathrm{pr}}\right)-\Delta A^{\mathrm{PA}}\left(\nu_{\mathrm{pu}}^{\mathrm{vis}},\nu_{\mathrm{pr}}\right). \tag{S5}$$

As discussed in the main text, we assume that the signal in our probe window is due solely to PA for excitation in the UV (i.e., at either 265 nm or 300 nm). This implies that $\sigma_i\left(\nu_{\mathrm{pr}}\right)=0$ for all i and for all probe frequencies in the probe window in our measurements (350 – 1100 nm) when the pump frequency is in the UV, $\nu_{\mathrm{pu}}^{\mathrm{UV}}$. In this case,

$$\Delta A\left(\nu_{\mathrm{pu}}^{\mathrm{UV}},\nu_{\mathrm{pr}}\right)=\Delta A^{\mathrm{PA}}\left(\nu_{\mathrm{pu}}^{\mathrm{UV}},\nu_{\mathrm{pr}}\right)\propto\sum_i N_i\sigma_i\left(\nu_{\mathrm{pu}}^{\mathrm{UV}}\right)\sigma_i'\left(\nu_{\mathrm{pr}}\right). \tag{S6}$$

The similar TA spectra (apart from the moving spectral hole) and lack of spectral dynamics suggest that the PA spectrum of the sample is the same regardless of excitation wavelength. We can therefore use the signal recorded with UV excitation to model the PA signal appropriate for any visible excitation wavelength. This allows the GSB signal contribution to be isolated, and eq. S5 becomes,

$$\Delta A^{\mathrm{GSB}}\left(\nu_{\mathrm{pu}}^{\mathrm{vis}},\nu_{\mathrm{pr}}\right)=\Delta A\left(\nu_{\mathrm{pu}}^{\mathrm{vis}},\nu_{\mathrm{pr}}\right)-s\Delta A\left(\nu_{\mathrm{pu}}^{\mathrm{UV}},\nu_{\mathrm{pr}}\right)\propto-\sum_i N_i\sigma_i\left(\nu_{\mathrm{pu}}^{\mathrm{vis}}\right)\sigma_i\left(\nu_{\mathrm{pr}}\right). \tag{S7}$$

In eq. S7, s is a scaling constant that makes the PA-only signal match the signal containing both PA and GSB contributions at frequencies where no bleaching is present. The GSB signal contribution calculated with eq. S7 yields an inverted, Gaussian-shaped profile centered about the pump frequency ν_b for all pump wavelengths in our study (Fig. 3 in the main text). We then make the simple assumption that the lineshape of every absorber is described by a Gaussian function of identical width, but centered about that absorber's unique center frequency, $\nu_{0,i}$. If the absorption cross section of every absorber is approximately the same, we can write,

$$\sigma_i\left(\nu_{\mathrm{pu}}^{\mathrm{vis}}\right)=\sigma\left(\nu_{\mathrm{pu}}^{\mathrm{vis}}-\nu_{0,i}\right), \tag{S8}$$

allowing eq. S7 to be recast as,

$$\Delta A^{\mathrm{GSB}}\left(\nu_{\mathrm{pu}}^{\mathrm{vis}},\nu_{\mathrm{pr}}\right)\propto-\sum_i N_i\sigma\left(\nu_{\mathrm{pu}}^{\mathrm{vis}}-\nu_{0,i}\right)\sigma\left(\nu_{\mathrm{pr}}-\nu_{0,i}\right). \tag{S9}$$

With the change of variables, $\nu_i'=\nu_{\mathrm{pu}}^{\mathrm{vis}}-\nu_{0,i}$, eq. S9 can be rewritten,

$$\Delta A^{\mathrm{GSB}}\left(\nu_{\mathrm{pu}}^{\mathrm{vis}},\nu_{\mathrm{pr}}\right)\propto-\sum_i N_i\sigma\left(\nu_i'\right)\sigma\left(\nu_i'-\nu_{\mathrm{pu}}^{\mathrm{vis}}+\nu_{\mathrm{pr}}\right). \tag{S10}$$

Given our assumption that the absorbers have similar cross sections and given that the absorption spectrum of eumelanin is approximately flat over the spectral region of the hole, the number density of absorbers is approximately the same per unit frequency over the width of the spectral hole. Then, N_i can be replaced by N, and the right-hand side of eq. S10 can be recognized as a discretized form of the auto-correlation of σ with itself,

$$\Delta A^{\mathrm{GSB}}\left(\nu_{\mathrm{pu}}^{\mathrm{vis}},\nu_{\mathrm{pr}}\right)\propto\left(\sigma\otimes\sigma\right)\left(\nu_{\mathrm{pu}}^{\mathrm{vis}}-\nu_{\mathrm{pr}}\right)=\left(\sigma\otimes\sigma\right)\left(\nu_{\mathrm{pr}}-\nu_{\mathrm{pu}}^{\mathrm{vis}}\right), \tag{S11}$$

where the symbol $\otimes$ stands for cross-correlation. Eq. S11, the main result of this simple model, predicts that the spectrum of the bleach contribution to the TA signal is equal to the autocorrelation of the Gaussian absorption band of one absorber with itself, centered about the (visible) pump frequency $\nu_{\mathrm{pu}}^{\mathrm{vis}}$. Cross-correlation is the same as autocorrelation for a symmetric function like a Gaussian, so the measured full-width at half-maximum (FWHM) of the hole in the GSB signal should be divided by the square root of two to estimate the FWHM of the lineshape function of a single absorber.

S.5. Procedure for subtracting out positive contributions to the transient absorption signals in DOPA melanin measured using 400, 500, and 600 nm excitation

Using the model in section S.4, the ground state bleach (GSB) spectra were isolated from the total transient absorption (TA) spectra measured using 400, 500, and 600 nm excitation. For each time delay, a linear combination of a negative Gaussian and the TA spectrum measured using 265 nm excitation was fit to the TA spectra measured using either 400, 500, or 600 nm excitation:

$$F(E,t) = -a_1(t)\exp\left(-0.5\left(\frac{(E-E_0(t))}{\text{FWHM}(t)/2.355}\right)^2\right) + a_2(t)\Delta A_{265\,nm}(E,t) \qquad \text{(S12)}$$

where E is photon energy, t is delay time, $a_1(t)$ and $a_2(t)$ are positive scaling factors, $E_0(t)$ and FWHM(t) are the peak center and FWHM of the Gaussian function, and $\Delta A_{265\text{ nm}}(E,t)$ is the transient absorption spectrum measured using 265 nm excitation. This procedure was followed for the TA spectra recorded using the magic angle and perpendicular pump-probe polarization geometries. Figure S4 shows examples of this procedure for several time delays.

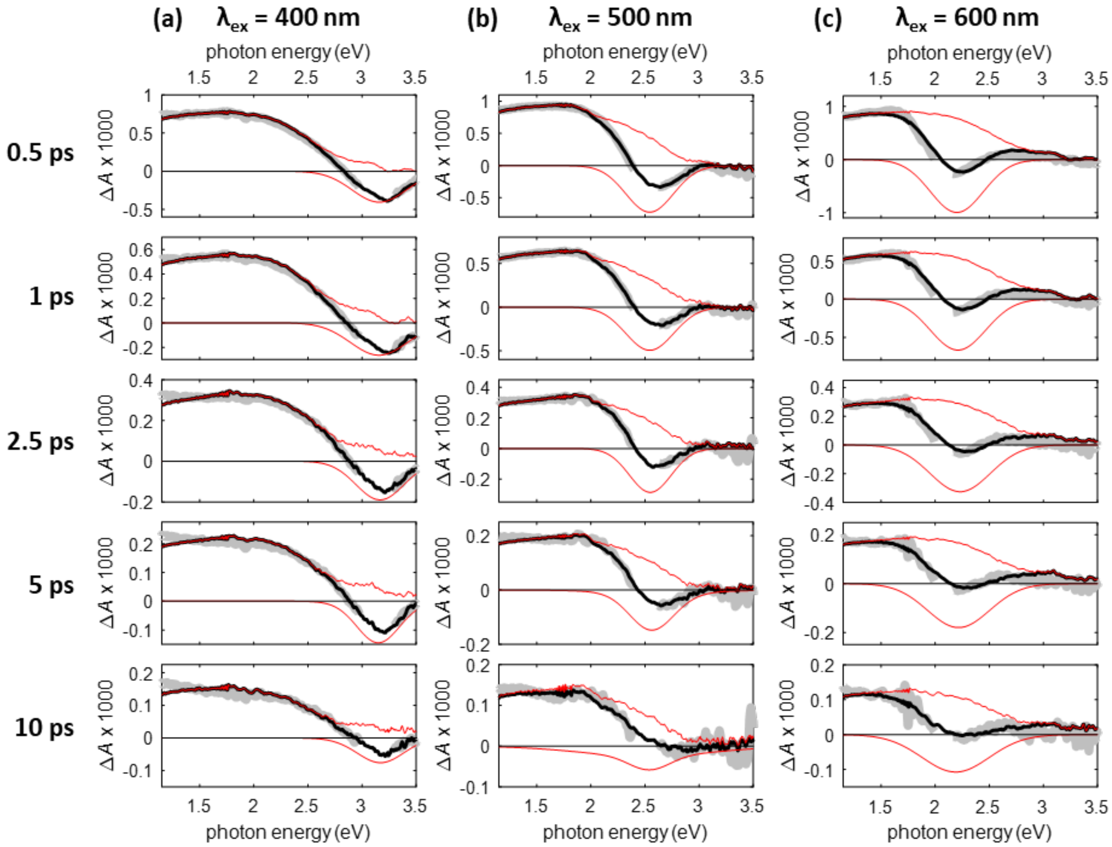

Figure S4. Spectral decomposition procedure illustrated for several time delays for the transient absorption spectra measured using 400, 500, and 600 nm excitation at the magic angle pump-probe polarization geometry (grey curves). The best fits are shown as black curves and the scaled basis functions as expressed in eq. S12 are shown as red curves.

The results of the decomposition procedure illustrated in Figure S4 are shown for TA spectra recorded using either the magic angle or perpendicular pump-probe polarization geometries in Figure S5. The isolated time-dependent GSB spectra for both polarization geometries are in close agreement.

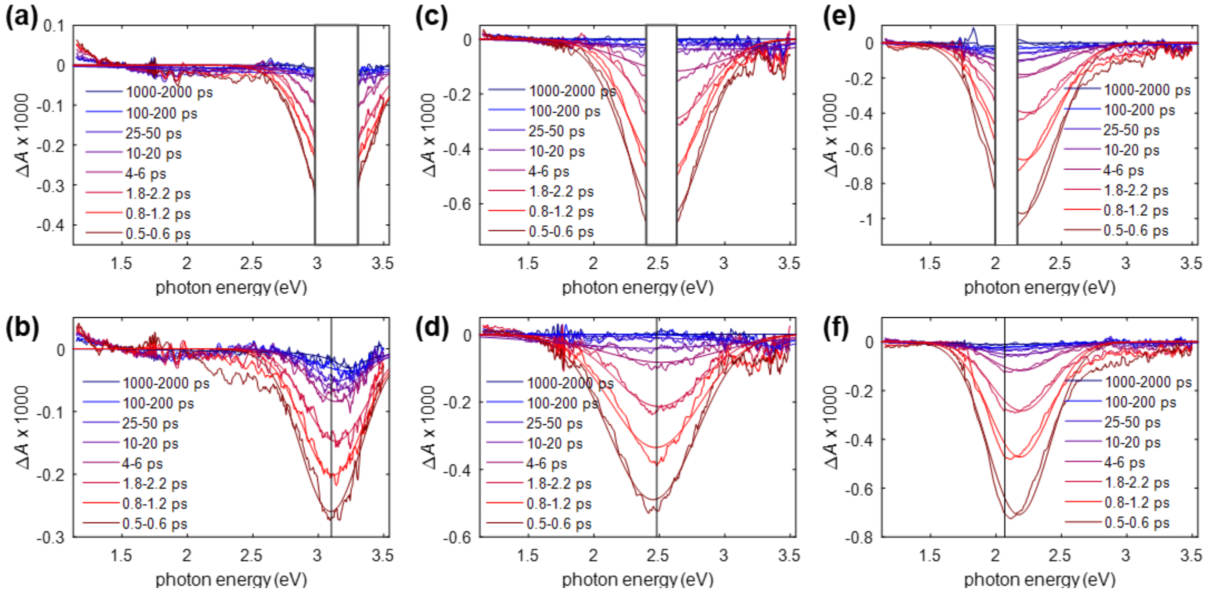

Figure S5. Decomposed time-resolved ground state bleach spectra of DOPA melanin recorded using magic angle pump-probe polarization and with 400, 500, and 600 nm excitation are shown in (a), (c), and (e), respectively. The spectra recorded using perpendicular pump-probe polarization and with 400, 500, and 600 nm excitation are shown in (b), (d), and (f), respectively.

From eq. S12, we obtain the FWHM of the negative Gaussian function describing the GSB feature. These values are summarized in **Table S1** for the TA spectra recorded using the visible excitation wavelengths.

Table S1. Best-fit parameter values for the FWHM of the ground state bleach spectrum of DOPA melanin at 1 ps, given for magic angle and perpendicular pump-probe polarizations.

**Excitation wavelength (nm)**	**Magic Angle FWHM (eV)^a**	**Perpendicular FWHM (eV)^a**
400	0.62 ± 0.06	0.54 ± 0.05
500	0.66 ± 0.07	0.87 ± 0.09
600	0.60 ± 0.06	0.56 ± 0.06

[a] Two sigma uncertainties are roughly estimated to be 10% of the FWHM value from the fits.

S.6. Time dependent anisotropy for 400 nm excitation of DOPA melanin

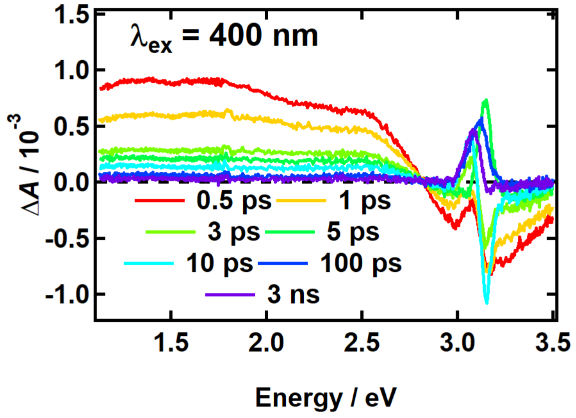

Figure S6. Transient absorption spectra of DOPA melanin excited at 400 nm at the indicated delay times using parallel pump-probe polarizations.

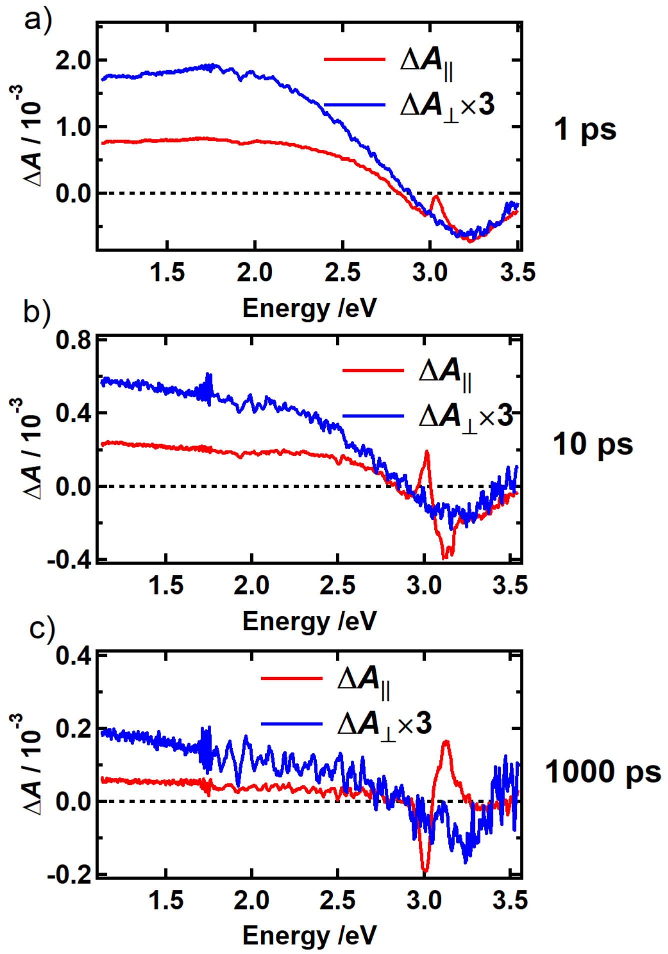

Figure S7. Comparison of the TA spectra for 400 nm excitation at a) 1 ps, b) 10 ps, and c) 1000 ps for parallel (red) and perpendicular (blue, multiplied by 3) pump-probe polarizations.

S.7. Modeling parameters for transient absorption kinetics at 950 nm

The transient absorption kinetics measured at 950 nm using 265, 300, 400, 500, and 600 nm excitation wavelengths were fit to a linear combination of a stretched exponential and power law as described in the main text. This model was convoluted with a Gaussian function of 200 – 250 fs FWHM representing the instrument response function and then used to fit the data. Two series of fits are shown: (Fit 1) The stretching parameter (β) is fixed for all fits; (Fit 2) β is fixed for all fits, while the stretched exponential lifetime (τ) and power law exponent (α) are fixed for the 300, 400, 500, and 600 nm excitation wavelengths to the values obtained from fitting the kinetics corresponding to 265 nm excitation. The best-fit curves are compared to the kinetic data for both cases in **Figure S8**. The quality of the fits are very good regardless of whether the τ and α values are fixed or not.

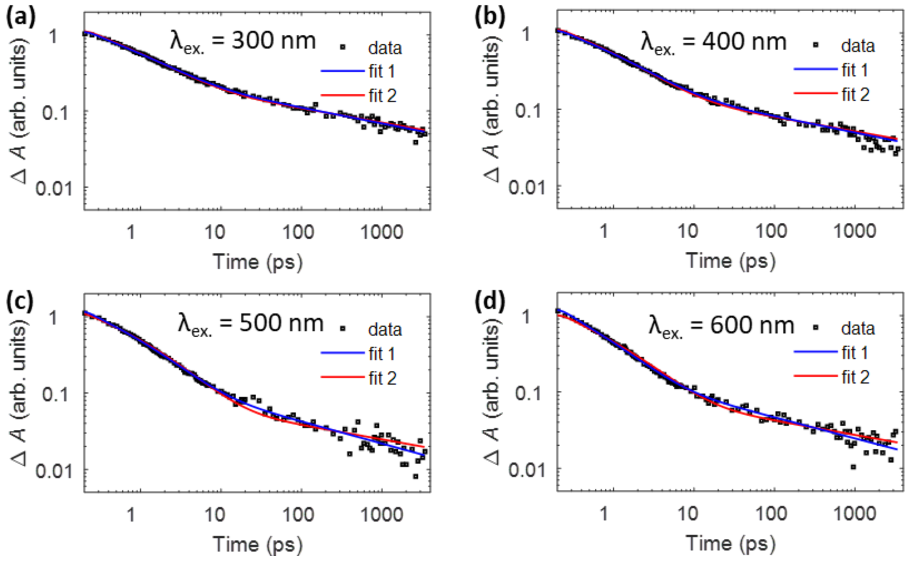

Figure S8. Comparison of the transient absorption kinetics to the best fits for **(a)** 300, **(b)** 400, **(c)** 500, and **(d)** 600 nm excitation. 'Fit 1' represents a fit in which only the β parameter is fixed at 0.33 (fit parameters listed in Table S2). 'Fit 2' represents a fit in which the β parameter is fixed at 0.33 and the τ and α parameters are fixed to the values obtained from fitting the kinetics measured using 265 nm excitation (fit parameters listed in Table S3).

The parameters obtained from both series of fits are summarized in **Table S2** – **S3.**

Table S2. Fit results for DOPA melanin transient absorption kinetics probed at 950 nm with excitation at the indicated wavelengths.

parameter	265 nm	300 nm	400 nm	500 nm	600 nm
$A_1 / (A_1 + A_2)$	0.87	0.88	0.91	0.96	0.96
$A_2 / (A_1 + A_2)$	0.13	0.12	0.087	0.045	0.043
τ (ps)	0.114	0.111	0.117	0.072	0.052
β	0.33 (fixed)	0.33 (fixed)	0.33 (fixed)	0.33 (fixed)	0.33 (fixed)
α	0.188	0.207	0.214	0.289	0.276

Table S3. Fit results for DOPA melanin transient absorption kinetics probed at 950 nm with fixed τ and α parameters and for excitation at 300, 400, 500, and 600 nm.

parameter	265 nm	300 nm	400 nm	500 nm	600 nm
$A_1 / (A_1 + A_2)$	0.87	0.90	0.93	0.97	0.96
$A_2 / (A_1 + A_2)$	0.13	0.10	0.068	0.029	0.037
τ (ps)	0.114	0.114 (fixed)	0.114 (fixed)	0.114 (fixed)	0.114 (fixed)
β	0.33 (fixed)	0.33 (fixed)	0.33 (fixed)	0.33 (fixed)	0.33 (fixed)
α	0.188	0.188 (fixed)	0.188 (fixed)	0.188 (fixed)	0.188 (fixed)

S.8. Isolated ground state bleach kinetics using 500 and 600 nm excitation

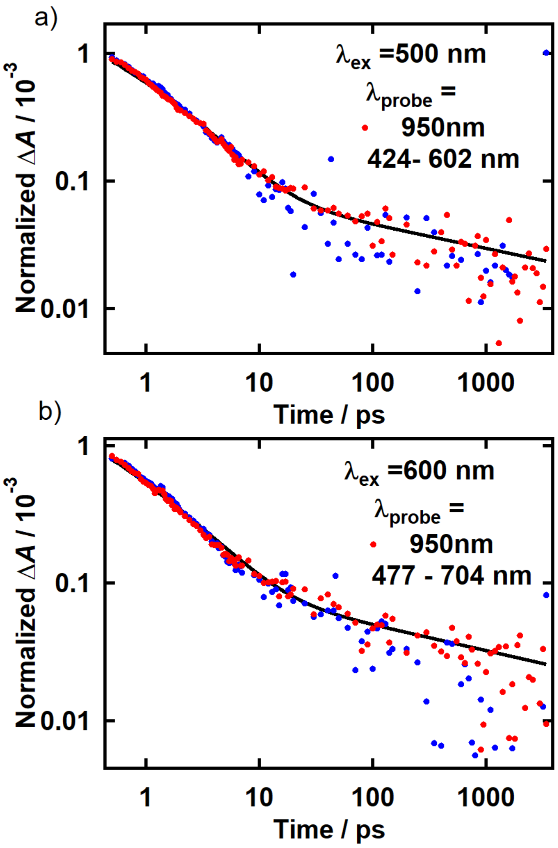

Figure S9. Comparison of normalized kinetics for the ground state bleach signal (blue markers, inverted data) and the the positive band for a) 500 nm and b) 600 nm excitation. The bleach signals were isolated as described in sections S4 and S5 and then averaged over the indicated wavelength range.

S.9. Size estimation of DOPA Melanin sp^2 domains from Raman bands

We find that DOPA melanin film has "stage 2" disorder in the scheme of Ferrari and Robertson[5] based on (1) The ~30 cm^{-1} downshift of the G band as wavelength is increased from 458 to 633 nm, (2) the peak center of the G band falls within the 1510 – 1600 cm^{-1} range, and (3) the intensity ratio of the D to G bands, I(D)/I(G), is appreciable and not close to zero. In this regime, Ferrari and Robertson have shown that I(D)/I(G) is related to the in-plane correlation length, L_a, of sp^2 domains by the equation,

$$\frac{I(D)}{I(G)} = C'(\lambda) L_a^2 , \tag{S13}$$

where the wavelength-dependent empirical parameter $C'(\lambda)$ equals 0.55 nm^{-2} when the wavelength, λ, equals 514 nm.[5] Using this equation, our the D:G peak height ratio of 0.55, measured using 514 nm, predicts an sp^2 domain length of ~1 nm in DOPA melanin.

S.10 Spectral hole burning of DOPA melanin and graphene oxide

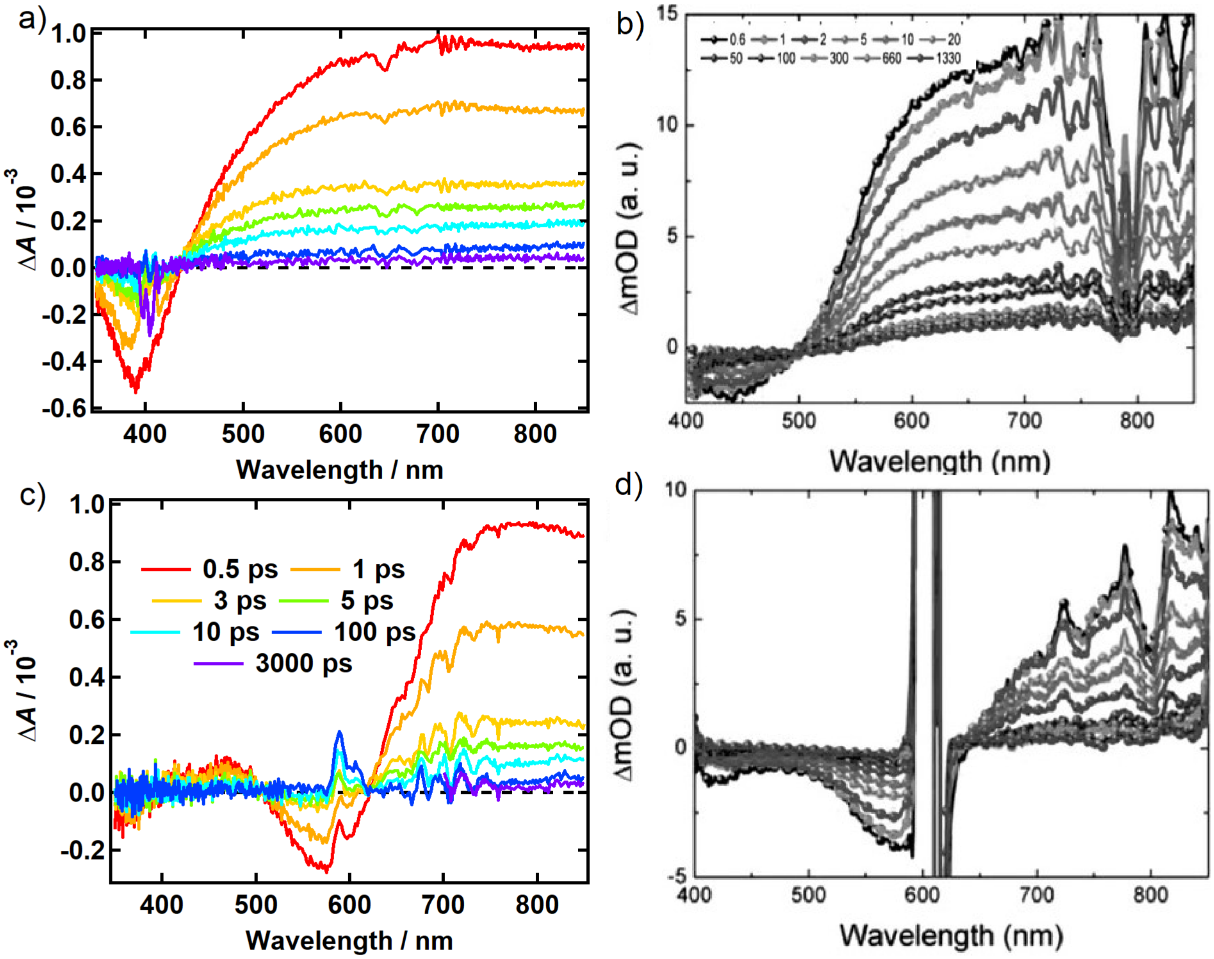

Figure S10. Comparison of the transient absorption spectra of DOPA melanin recorded in this study (a,c; magic angle pump-probe polarizations) with ones from graphene oxide (b,d; delay times are indicated in the legend in panel b) with 400 nm (a,b) and 600 nm excitation (c,d). The graphene oxide data in panels b and d are adapted with permission from L. Wang et al., *Adv. Mater.* 2013, **25**, 6539-6545, copyright WILEY-VCH Verlag GmbH & Co. KGaA, Weinheim.

S.11 References

1. A. B. Mostert, K. J. P. Davy, J. L. Ruggles, B. J. Powell, I. R. Gentle and P. Meredith, *Langmuir*, 2010, **26**, 412-416.
2. C. Grieco, F. R. Kohl, Y. Zhang, S. Natarajan, L. Blancafort and B. Kohler, *Photochem. Photobiol.*, 2019, **95**, 163-175.
3. C. Grieco, Jennifer M. Empey, F. R. Kohl and B. Kohler, *Faraday Discuss.*, 2019, **216**, 520-537.
4. B. Kohler, in *Ultrafast Dynamics at the Nanoscale: Biomolecules and Supramolecular Assemblies*, eds. I. Burghardt and S. Haacke, Pan Stanford Publishing Pte Ltd, Singapore, 2017, DOI: 10.4032/9789814745345, pp. 3-64.
5. A. C. Ferrari and J. Robertson, *Phys. Rev. B*, 2000, **61**, 14095-14107.
6. L. Wang, H. Y. Wang, Y. Wang, S. J. Zhu, Y. L. Zhang, J. H. Zhang, Q. D. Chen, W. Han, H. L. Xu, B. Yang and H. B. Sun, *Adv Mater*, 2013, **25**, 6539-6545.
