# Supplementary material for: Ultrafast spectral hole burning reveals the distinct chromophores in eumelanin and their common photoresponse
Source: Chem Sci. 2019 Dec 18;11(5):1248–59. doi: 10.1039/c9sc04527a (PMC8148383; doi:10.1039/c9sc04527a)
Supplement: SC-011-C9SC04527A-s002 [file SC-011-C9SC04527A-s002.pdf]

Electronic Supplementary Material (ESI) for Chemical Science.
This journal is © The Royal Society of Chemistry 2019

The Colours in Black : An Ultrafast Spectral Study of Eumelanin

Eumelanin is a brown-black pigment that dissipates harmful solar light and prevents cell damage in many organisms

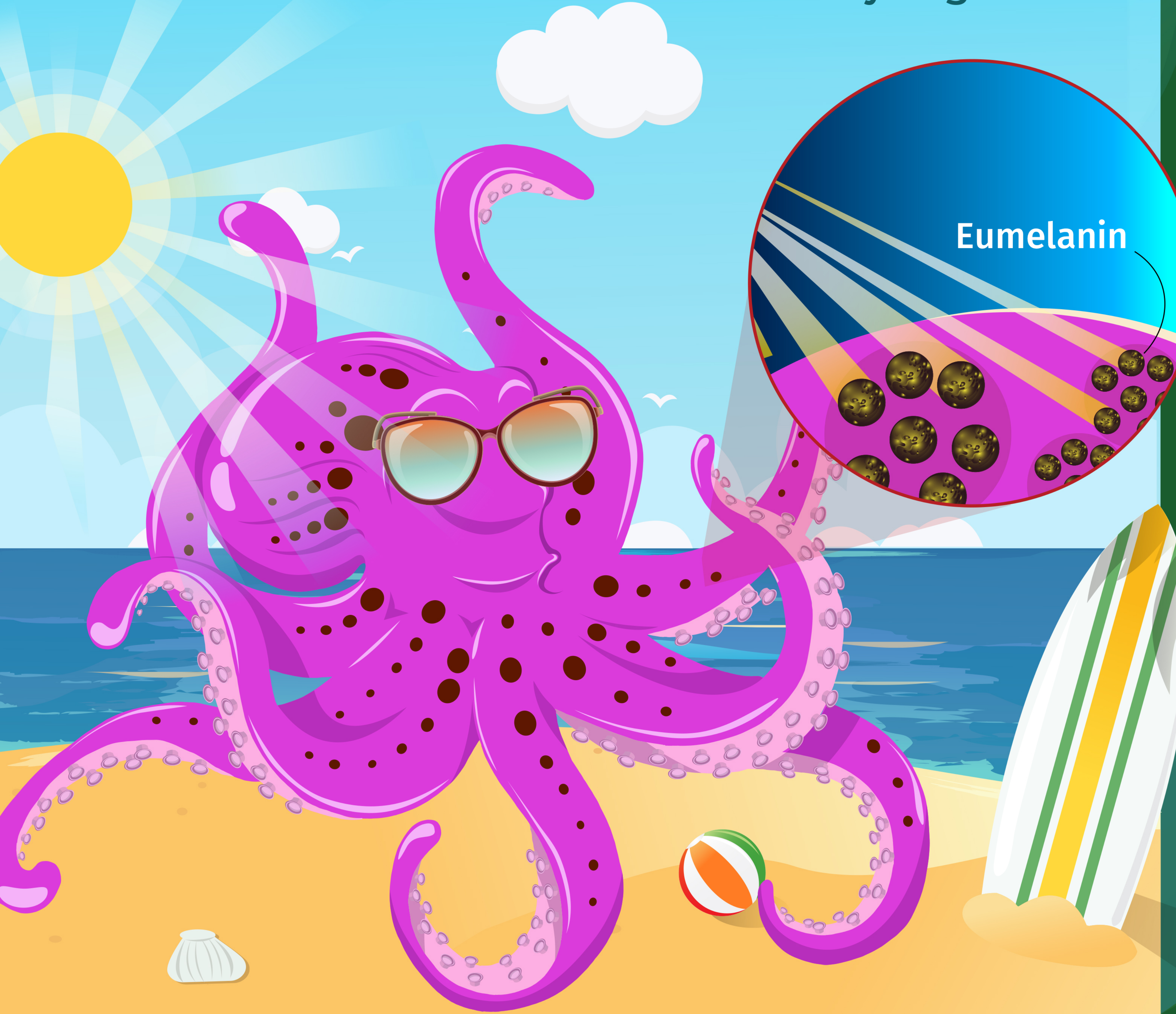

The chromophores that help eumelanin absorb light over a broad spectrum and their characteristics remain unclear

What are the light absorption characteristics of chromophores in eumelanin?

Using ultrafast spectral hole burning, the excited state behaviour of synthetic eumelanin was studied

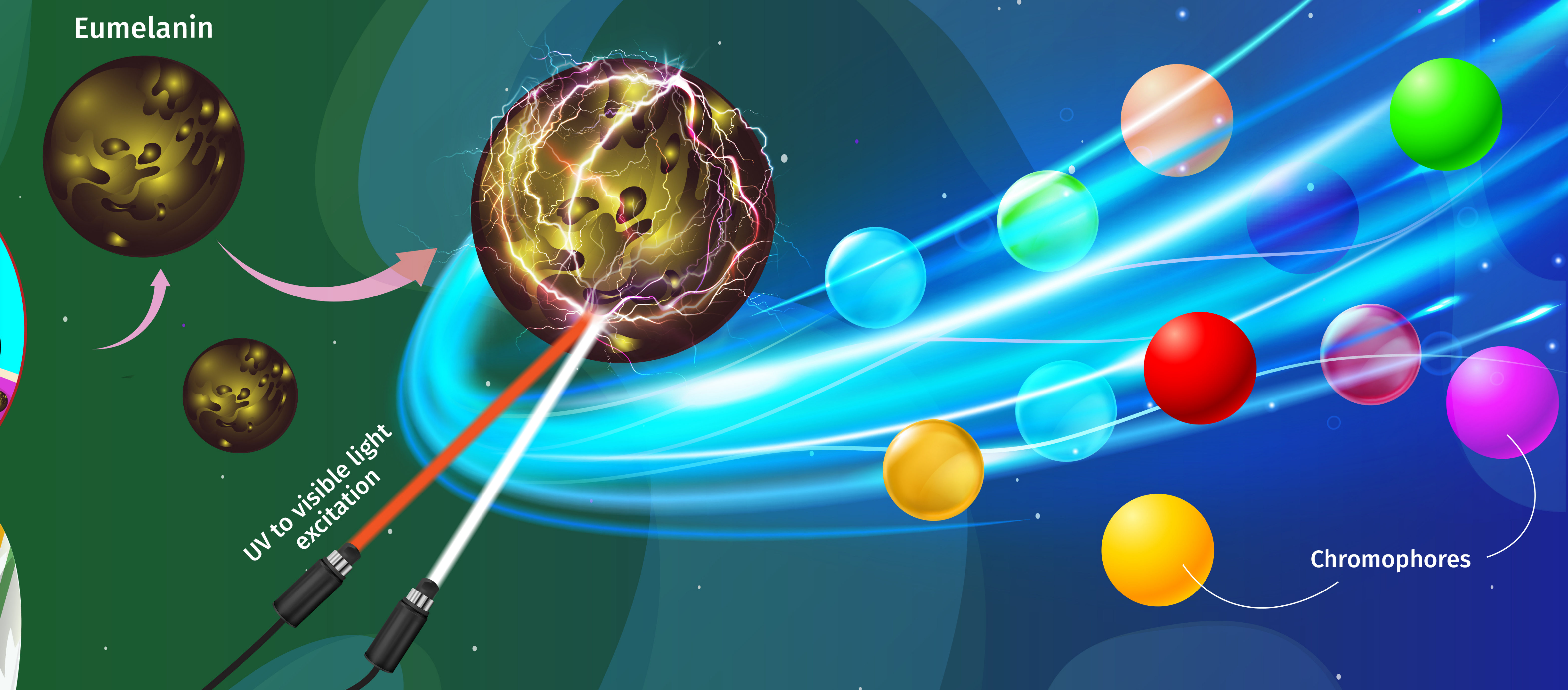

Eumelanin has a large number of coloured chromophores which share excited state dynamics with disordered carbon nanomaterials

Understanding chromophore dynamics of eumelanin will be invaluable for developing structure–function models for many carbonaceous nanomaterials

Chemical Science
PICK OF THE WEEK
Ultrafast spectral hole burning reveals the distinct chromophores in eumelanin and their common photoresponse
Kohler *et al.* (2019) | DOI: 10.1039/C9SC04527A
ROYAL SOCIETY OF CHEMISTRY
